# Supra-3‑V Nonaqueous Redox-Flow Batteries Based on Simple Terephthalonitrile Anolytes

**DOI:** 10.1021/acsaem.5c01949

**Published:** 2025-10-06

**Authors:** Nicolas Daub, Xiaotong Zhang, Nico J. L. van Rijswijk, Piotr de Silva, René A. J. Janssen

**Affiliations:** † Molecular Materials and Nanosystems and Institute for Complex Molecular Systems, 3169 Eindhoven University of Technology, P.O. Box 513, 5600 MB Eindhoven, The Netherlands; ‡ Department of Energy Conversion and Storage, 5205 Technical University of Denmark, Anker Engelunds Vej 301, 2800 Kongens Lyngby, Copenhagen, Denmark; § Dutch Institute for Fundamental Energy Research, De Zaale 20, 5612 AJ Eindhoven, The Netherlands

**Keywords:** nonaqueous redox-flow battery, anolyte, terephthalonitrile, density functional theory, cycling stability

## Abstract

Organic molecules for nonaqueous redox-flow batteries tend to become increasingly complex because, for practical applications, they have to fulfill several requirements in terms of redox potential, solubility, and stability. Implementing these functionalities in the design of materials often results in an undesirable high synthetic complexity, which reduces the feasibility for large-scale applications. Considering redox potential, solubility, stability, and low synthetic complexity as important design considerations, we investigated the suitability of alkyl-substituted terephthalonitriles for use as anolytes in redox-flow batteries. These derivatives can be synthesized in two steps. With one ethyl substituent, the stability is very limited because the reduced anolyte deprotonates the solvent, which then reacts with the neutral anolyte. Experiments and density functional theory calculations show that this reaction can be slowed down by introducing two alkyl substituents. In combination with dialkoxy-substituted benzene derivatives as the catholyte, 2,5-dialkylterephthalonitriles achieve >3 V flow batteries that exhibit capacity retention of >99.8% cycle^–1^ and energy efficiencies of up to 77% at a current density of 40 mA cm^–2^. With these metrics, 2,5-dialkylterephthalonitriles outperform many previously reported flow batteries using benzene-based anolytes, but for future practical application, solubility and long-term stability need to be further enhanced.

## Graphical abstract

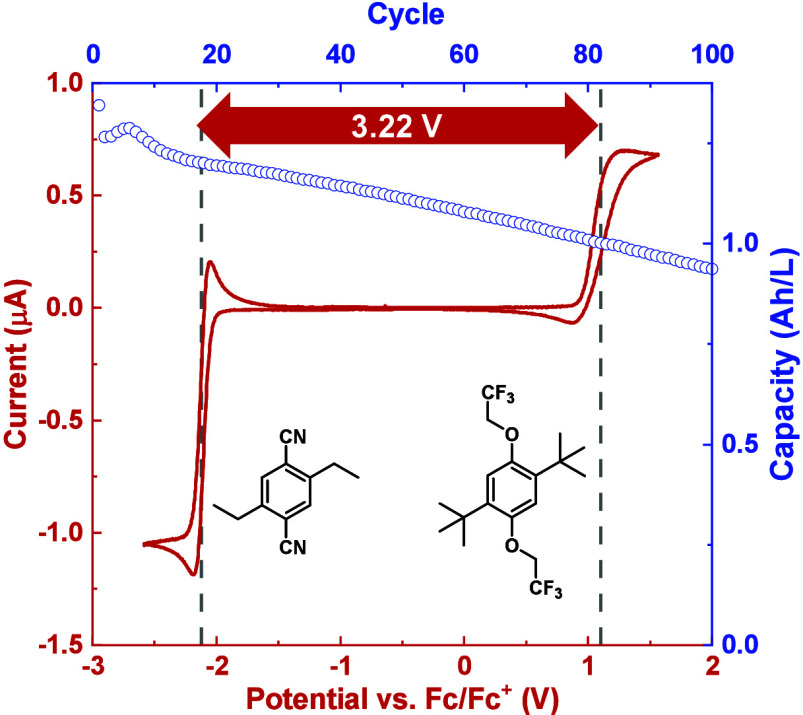

## 1 Introduction

Implementation of intermittent renewable energy sources, such as wind and solar, in the energy grid will require the use of large-scale energy storage to mitigate the discrepancies between energy production and demand. Nonaqueous organic redox-flow batteries from abundant carbon-based materials can provide a sustainable solution. In an organic redox-flow battery, the redox-active species are dissolved or suspended in a solvent with a supporting electrolyte, forming an anolyte and a catholyte.^1^ These solutions flow through a reactor, where they are passed over porous carbon electrodes that mediate electron-transfer reactions to charge or discharge the solutions. The two half-cells in the reactor are separated by a membrane or a porous separator, which allows for a compensating ion flux that sustains the electrical current of the external circuit.^2^ The advantage of a redox-flow battery is that the capacity is determined by the size of the electrolyte storage tanks and the power output is determined by the size of the reactor. These metrics can be independently scaled, allowing for massive energy storage without the need for expensive large-surface-area electrodes.^3^

Current organic redox-flow battery technology, however, has not reached a mature enough level for implementation, and many scientific questions still need to be answered. Specifically, redox-active materials need to be developed that simultaneously deliver high cell voltages and high energy density in combination with good energy efficiency (EE), cyclability, and stability.^4–6^ Additionally, developing a cost-effective, scalable synthesis route is crucial for the upscaling of all-organic redox-flow batteries. This might involve using cheaper starting materials or minimizing the number of synthesis steps.

To increase the cell voltage, anolytes with deep reduction potentials are required. While benzophenone-based anolytes possess reduction potentials at around −2.3 V vs ferrocenium/ferrocene (Fc^+^/Fc), their moderate stability with a decay of 0.4–1.0% cycle^–1^ and cell voltages below 3 V (Table S1) leave room for improvement.^7–11^ In the search for stable, soluble, and deep-reduction potential anolytes, we herein consider the suitability of terephthalonitrile derivatives (1,4-dicyanobenzenes) as anolytes for redox-flow batteries because they can be synthesized via simple procedures using affordable starting materials.^12,13^ Terephthalonitriles are known to form relatively persistent radical anions upon single-electron reduction at potentials below −2 V vs Fc^+^/Fc and have previously been used as precursors in electrochemical radical coupling reactions^14^ and as electrode material in rechargeable lithium-ion batteries.^15^

Here, we studied 2-ethylterephthalonitrile (ET),^16^ 2,5-diethylterephthalonitrile (DET), and 2,5-di-*tert*-butylterephthalonitrile (DTBT) (Figure 1) and determined their electrochemical properties and stability in the reduced state to investigate whether the dicyanobenzene motif is viable for use as an anolyte in redox-flow batteries. The presence of two alkyl substituents in DET and DTBT successfully suppressed a degradation reaction that occurred for ET in which the reduced anolyte deprotonated the solvent, which then reacted with the anolyte. Density functional theory (DFT) calculations confirm that steric hindrance by alkyl substituents increases the activation energy for the forward and backward reactions. Redox-flow batteries with DET or DTBT as the anolyte in combination with 1,4-di-*tert*-butyl-2,5-bis­(2,2,2-trifluoroethoxy)­benzene (DBBTFB) or 2,5-di-*tert*-butyl-1,4-bis­(2-methoxyethoxy)­benzene (DBBB) (Figure 1) as the catholyte provided high cell voltages (2.76–3.22 V), a capacity retention (99.4–99.9%) per cycle, and energy efficiencies of 72–80% at current densities of 30–40 mA cm^–2^. While this represents a similar and improved performance compared to known deep-reduction potential anolytes, the solubility and stability under cycling conditions remain to be improved for practical applications.

**Figure 1:**
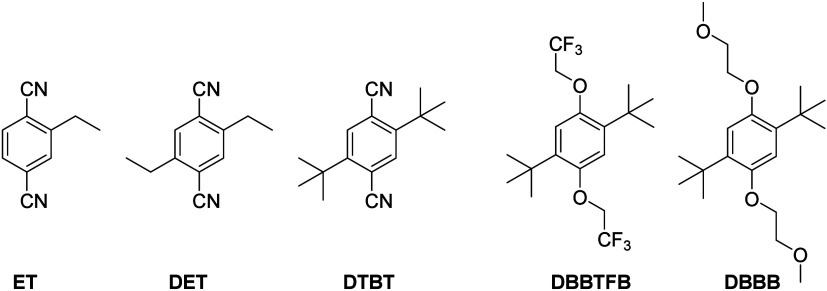
Structures of anolytes and catholytes used. Anolytes: ET, DET, and DTBT. Catholytes: DBBTFB and DBBB.

## 2 Experimental Section

### 2.1 Materials

Acetonitrile (99.9%, Extra Dry over Molecular Sieve, AcroSeal, Thermo Fisher Scientific) and tetrabutylammonium hexafluorophosphate (TBAPF_6_, ≥99.0%, for electrochemical analysis, Sigma-Aldrich/Merck) were used as received. 2-Ethylterephthalonitrile (ET) and 2,5-di-*tert*-butyl-1,4-bis­(2-methoxyethoxy)­benzene (DBBB) were acquired from abcr GmbH. ET was recrystallized twice from heptane before use. 2,5-Diethylterephthalonitrile (DET) was synthesized from 1,4-dibromo-2,5-diethylbenzene using a Rosenmund–von Braun cyanation reaction as described previously.^17,18^ The synthesis of 2,5-di-*tert*-butylterephthalonitrile (DTBT) has been reported previously,^19^ but it was instead prepared by reacting 1,4-di-*tert*-butylbenzene with bromine using iodine as a catalyst, followed by a cyanation reaction. 1,4-Di-*tert*-butyl-2,5-bis­(2,2,2-trifluoroethoxy)­benzene (DBBTFB) was synthesized based on the procedure of Bheemireddy et al.^20^ Details of the synthetic procedures and molecular characterization can be found in the Supporting Information.

### 2.2 Electrochemical Characterization

Stock solutions of the supporting electrolyte in acetonitrile were prepared in a nitrogen-filled glovebox at 27 °C. Cyclic voltammetry (CV), H-cell, and battery cycling experiments were conducted with a Biologic VSP potentiostat with the samples in a nitrogen-filled glovebox at 27 °C. The stability under charging and discharging was analyzed using an H-cell with a P5 porous glass frit separator and stirred compartments. A custom-made cell was used for redox-flow battery cycling,^21,22^ consisting of graphite charge-collecting plates with two layers of a carbon felt electrode (GDL 29 AA, 180 μm, 2.55 cm^2^) each, separated by a porous separator (Daramic 175, donated by Daramic LLC). The carbon felts and membrane were encapsulated with Gore-Tex ePTFF gaskets (Eriks). Assembly was done in ambient. A peristaltic pump (Cole Parmer) was used to create a flow rate of 25 mL min^–1^, employing Masterflex C-flex ultrapump tubing (Cole Parmer) between the flow cell and reservoirs, which were filled with solutions containing the redox-active materials and electrolyte salt. Before the measurement was started, the cell was pretreated by flowing the solution through the cell until the potentiostatic electrochemical impedance spectroscopy (PEIS) measurement did not change anymore. Galvanostatic charge–discharge cycling was performed using the currents and cutoff potentials reported. PEIS measurements were performed at various stages of charge from 200 kHz to 100 Hz using a 10 mV sine perturbation. A polarization measurement was collected at the full state-of-charge (SoC) and ranged from −25.5 to −400 mA. The EE of the batteries was determined by the ratio of the time-integrated output and input power densities during discharging and charging over each cycle.

To assess the stability of reduced anolytes over time, they were electrochemically reduced in an H-cell at 50 mM concentration in acetonitrile containing TBAPF_6_ (200 mM). Next, the reduced anolyte solution was removed from the H-cell and stored in a vial. At specific times, aliquots were removed from the vial and measured outside the glovebox under a continuous nitrogen stream with a PerkinElmer Lambda 1050 UV–vis–near-IR absorption spectrophotometer using a 0.2-mm-path-length quartz cuvette.

The CV curves of the anolyte and catholyte compartments were recorded before and after cycling with a micro-CV setup comprising a three-electrode system consisting of a glassy carbon disk working electrode (0.0079 mm^2^ surface area), an Ag^+^/Ag reference electrode with 0.01 M AgBF_4_ (Sigma-Aldrich) in acetonitrile, and a platinum disk counter electrode. Ferrocene (Fc^+^/Fc) was used as an internal reference.

## 3 Results and Discussion

### 3.1 Electrochemical Properties

CV curves recorded in acetonitrile solutions containing tetrabutylammonium hexafluorophosphate (TBAPF_6_) as the electrolyte revealed chemically reversible reduction waves for the three derivatives at potentials below −2 V vs Fc^+^/Fc (Figure 2).

**Figure 2:**
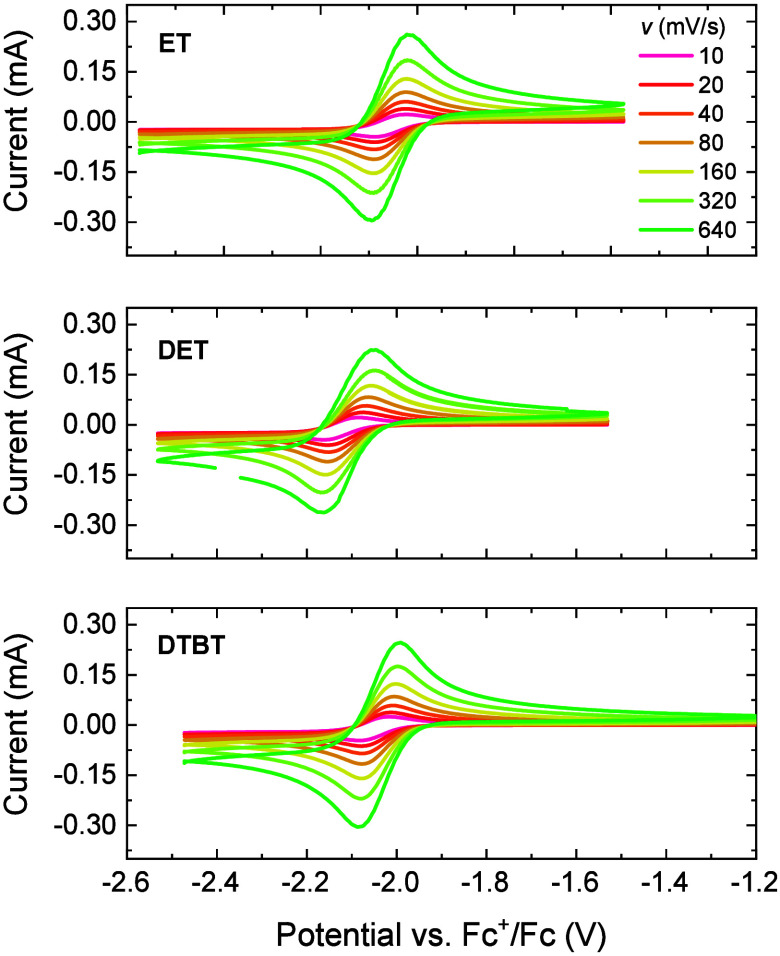
Electrochemical characterization of the anolytes. CV curves of 5 mM solutions of ET, DET, and DTBT in acetonitrile containing TBAPF_6_ (200 mM) were recorded at scan rates from 10 to 640 mV s^–1^.

CV at different scan rates (*v*) was used to determine the diffusion coefficients (*D*) via the Randles–Ševčik equation^23^ and the electron-transfer rates (*k*_0_) following the Nicholson method.^24^ The data are shown in Figure S1, and the values are summarized in Table 1 for dilute solutions of ET, DET, and DTBT in acetonitrile. Mass-transfer processes and electrokinetics play an important role in redox-flow battery applications.^1^ Fast diffusion and electron-transfer processes are vital for achieving high current densities and minimizing overpotentials. The *D* and *k*_0_ values are all on the same order of magnitude and higher than those for similar molecules reported for flow batteries and are considered appropriate for redox-flow battery applications.^25,26^

**Table 1:** Solubility and Electrochemical Characteristics of ET, DET, and DTBT

compound	*E*_1/2_ vs Fc^+^/Fc (V)	solubility in acetonitrile (mol L^–1^)	solubility in 1 M TBAPF_6_ (mol L^–1^)	*i*_pc_/*i*_pa_ at 80 mV s^–1^	*D* (cm^2^ s^–1^)	*k*_0_ (cm s^–1^)
ET	–2.05	1.3	1.0	1.03	2.2 × 10^–5^	5.0 × 10^–2^
DET	–2.12	0.5	0.35	1.01	2.0 × 10^–5^	2.6 × 10^–2^
DTBT	–2.05	0.2	0.05	1.01	2.0 × 10^–5^	2.2 × 10^–2^

Another critical parameter for flow batteries is the solubility. While ET exhibited a solubility of 1 M in acetonitrile containing TBAPF_6_ (1 M) as the supporting electrolyte salt, the solubilities of DET and DTBT were lower at 0.35 and 0.05 M (Table 1), limiting the volumetric capacity to 9.4 and 1.3 Ah L^–1^. TBAPF_6_ (1 M) was used because this would be required for real-world applications.^27^ At a battery voltage of 3 V, DET could possibly reach an energy density of 14.1 Wh L^–1^, which is below the range of commercial aqueous vanadium redox-flow batteries (25–35 Wh L^–1^).^28^ The decreasing solubility on going from ET to DET and DTBT shows that (branched) aliphatic groups decrease solubility in the polar acetonitrile electrolyte. Substitution with oligo­(ethylene glycol) side chains is known to dramatically increase the solubility of organic redox-active compounds in polar electrolyte solutions and provides an opportunity to increase the volumetric capacity in the future.^21,22,29–33^

### 3.2 Stability of the Charged State

When the terephthalonitriles are electrochemically reduced to the corresponding radical anions, new peaks and shoulders appear in the UV–vis absorption spectrum. The changes upon the stepwise reduction of ET to ET^•–^ are shown in Figure S2. Most prominent are a new absorption band at ca. 350 nm, together with two smaller peaks near 410 and 440 nm. Furthermore, a low-intensity broad band appears between 650 and 950 nm. The new peaks agree with the spectrum reported for the radical anion of terephthalonitrile, which exhibits a strong peak at 344 nm with a higher energy shoulder, two less intense bands at 395 and 428 nm, and a low-energy transition at 880 nm.^34^ The strong peak at 344 nm has been assigned to a superposition of two allowed transitions: one involving the excitation of an electron from the highest doubly occupied molecular orbital (π) to the singly occupied orbital [π*­(S)] and another from π*­(S) to higher-lying (diffuse) unoccupied π* orbitals. The lower-energy transitions have been attributed to excitation from π*­(S) to a range of π* orbitals with localized (880 nm) or Rydberg character (395 and 428 nm). For DET and DTBT, the UV–vis spectra of the radical anions are very similar.

The intrinsic stability of the radical anions was monitored by recording UV–vis absorption spectra of ET^•–^, DET^•–^, and DTBT^•–^ over time (Figure 3). as described in the Experimental Section. In each case, the losses are gradual, but after 13 days, the solution of ET^•–^ had lost all of its initial charge, as inferred from the loss of the absorption bands at 347 and 437 nm (Figure 3a). Figure 3d shows the decay of the normalized absorption at 437 nm of ET^•–^ versus time. A comparison of the spectrum observed after 13 days to that of neutral ET reveals several differences (Figure 3a). The intensity of the absorption bands at 202, 240, and 249 nm increased, and a new broad peak at ca. 260 nm appeared. The low-intensity peaks at 289 and 298 nm can still be discerned. A new peak at 392 nm arises. This shows that the ET^•–^ radical anion degrades rather than being reoxidized to neutral ET. Parts b and c of Figure 3 show the UV–vis spectra of DET and DTBT and the corresponding DET^•–^ and DTBT^•–^ radical anions directly after preparation and their temporal evolution over several days of storage. After 20 days, more than 40% is still in the charged state, demonstrating improved stability of DET^•–^ and DTBT^•–^ compared to ET^•–^. Importantly, the loss of DET^•–^ and DTBT^•–^ with time coincides with partial recovery of the UV–vis spectra of neutral DET and DTBT. This indicates that the loss of signal is primarily the result of discharging rather than the extensive formation of degradation products, as found for ET. This can also be seen from the fact that, for the aged DET^•–^ and DTBT^•–^ samples, the shoulder formed at ca. 260 nm in the case of aged ET^•–^ is much less pronounced. Likewise, the new peak at 392 nm arising for aged ET^•–^ is not clearly visible for the aged samples of both DET^•–^ and DTBT^•–^.

**Figure 3:**
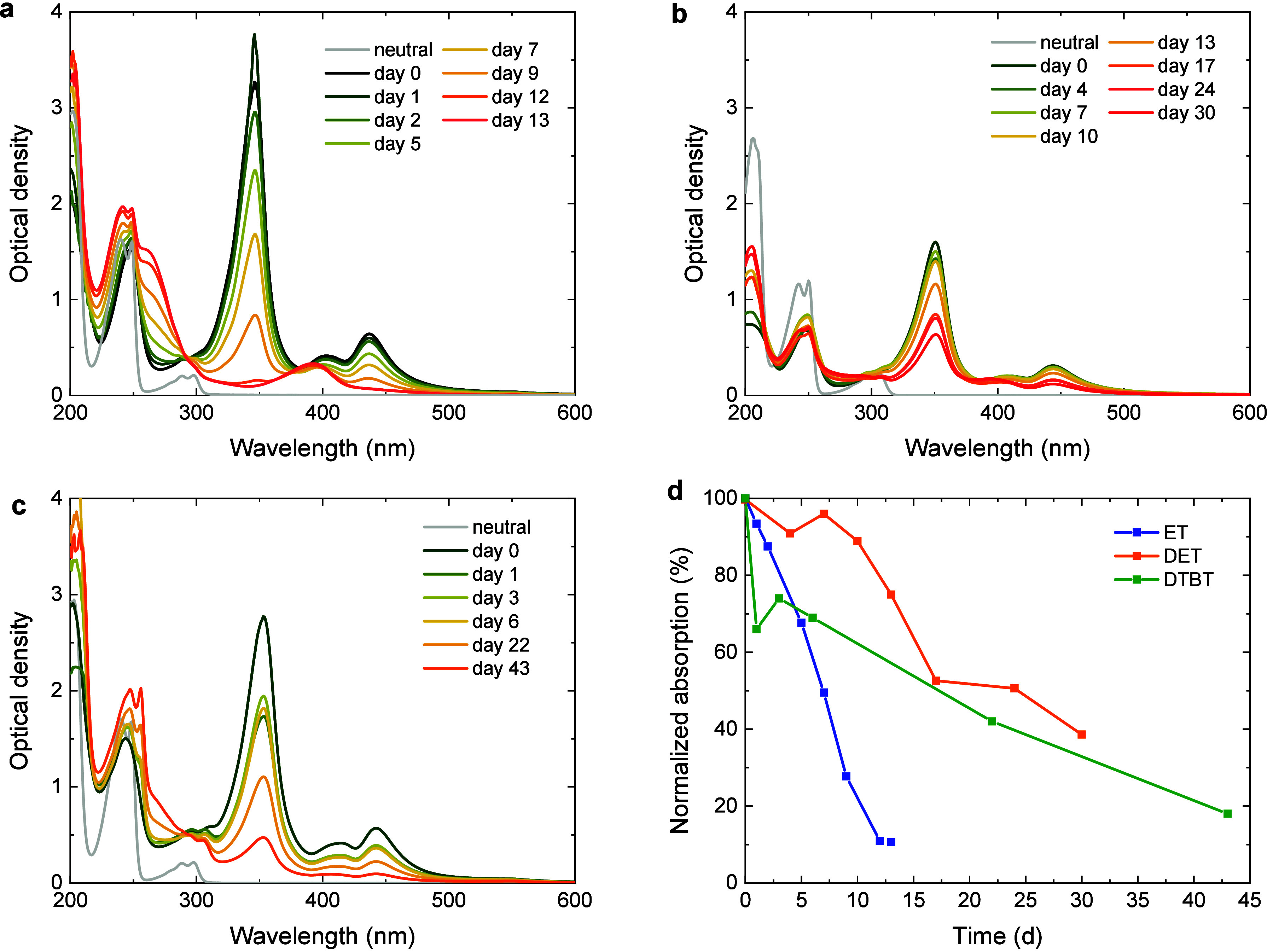
Temporal evolution of the UV–vis spectra of fully charged solutions of radical anions prepared by bulk electrolysis. Spectra were recorded for 50 mM solutions in acetonitrile containing TBAPF_6_ (200 mM): (a) ET; (b) DET; (c) DTBT. (d) Capacity retention vs time measured as the OD at 437 nm for ET, at 444 nm for DET, and at 354 nm for DTBT from the spectra in panels a–c.

The enhanced stability of DET and DTBT over ET is also evidenced from long-term electrochemical cycling data. Figure S3 shows the CV curves over 100 cycles for the three anolytes and reveals a retention of 95.6% for ET and 100% for DET and DTBT.

To further study the underlying degradation mechanism, a second shelf-life study of reduced ET was performed under the same conditions. ^1^H NMR after ET^•–^ was stored for 6 days revealed that no starting material was left (Figure S4a). A closer look at the NMR signals indicates the formation of products with new signals between 4.0 and 4.5 ppm and an upfield shift of the signals of the aromatic protons, which hints toward the substitution of one nitrile group with a less electron-withdrawing group. In fact, at least three new products were formed, as can be seen from the signals of aromatic protons between 7.3 and 8.1 ppm (Figure S4b). Part of the crude mixture was subjected to column chromatography, which resulted in a rather pure fraction that could be isolated. Gas chromatography–mass spectrometry (GC–MS) indicated a mass of *m*/*z* = 197 amu, corresponding to an adduct of ET with acetonitrile. Analysis by ^1^H and ^13^C NMR suggests that the product is (*Z*)-4-(1-amino-2-cyanovinyl)-2-ethylbenzonitrile (Figure 4a). Characteristic peaks of the vinylic and amino protons are found at δ = 4.29 and 4.97 ppm in the ^1^H NMR, with the corresponding signals of the vinyl carbons at 66.0 and 159.6 ppm in the ^13^C NMR (Figure S5). These characteristics agree with those of 3-amino-3-phenylacrylonitriles described by Beckmann et al.^35^ To confirm the degradation reaction shown in Figure 4a, ET was reacted with acetonitrile in toluene using potassium *tert*-butoxide (*t*-BuOK) as a base, following the procedure reported by Beckmann et al. The isolated product consisted of the *Z* and *E* isomers of 4-(1-amino-2-cyanovinyl)-2-ethylbenzonitrile, and its ^1^H NMR spectrum showed excellent agreement with the resonances of two of the three new products in the degraded sample of ET^•–^ (Figure S6).

**Figure 4:**
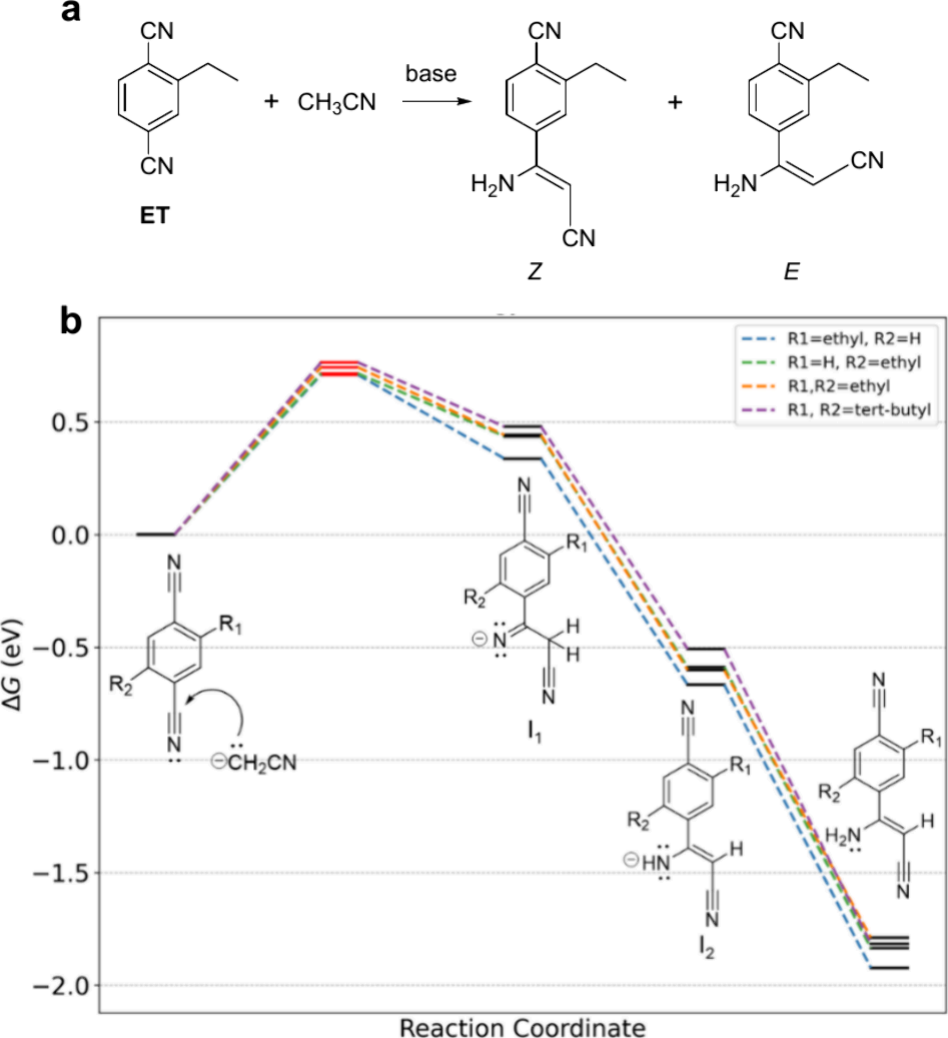
Formation of (*E*/*Z*)-4-(1-amino-2-cyanovinyl)-2-ethylbenzonitrile from ET and CH_3_CN in the presence of a strong base. (a) Overall reaction. Note that different regioisomers may be formed in this reaction. (b) Free energy profile for the reaction pathway of ET, DET, and DTBT with CH_2_CN^–^. Note that, for ET, nucleophilic attack of CH_2_CN^–^ on a nitrile group can result in two different regioisomers. The transition states are marked by short red lines, while intermediates and final products are represented by black lines.

Column chromatography also afforded another, less pure, product with *m*/*z* = 159 amu, i.e., 3 amu more than ET itself. The ^1^H and ^13^C NMR spectra of this product are shown in Figure S7. Numerous small impurity signals are seen, but further purification was not possible due to a very low isolated amount. Several possible products with *m*/*z* in the range from 156 to 160 amu were considered (Figure S8). The ^1^H NMR spectrum excludes products with aldehyde or imine groups because the expected =CH–Ar signals at 10.0 or 8.5 ppm were not found. Also, products with methylamino (−NHCH_3_) or aminomethyl (−CH_2_NH_3_) substituents are unlikely because the ^1^H NMR lacked expected singlets at 2.7 and 4.3 ppm for the methylene and methyl protons, respectively, and *m*/*z* = 160 amu is 1 amu too high. The best correspondence was found for 3,4-diethylbenzonitrile (*m*/*z* = 159.1 amu), where all expected signals could be identified in ^1^H and ^13^C NMR (Figure S7), leaving only a few minor impurity signals without a clear assignment. Together with the *Z* and *E* isomers of 4-(1-amino-2-cyanovinyl)-2-ethylbenzonitrile, the formation of 3,4-diethylbenzonitrile identifies the three main degradation products found for ET^•–^ (Figure S6).

The formation of functionalized 3-amino-3-phenylacrylonitriles from the corresponding benzonitriles is known to occur via an addition reaction with the acetonitrile anion prepared from acetonitrile with a strong base,^36,37^ and we assume that the ET^•–^ radical anion acts as the strong base. We note that this mechanism requires the presence of a neutral ET. We consider the following steps conceivable for its formation from ET^•–^:^38^

1 $${\mathrm{ET}}^{•-}+{\mathrm{CH}}_{3}\mathrm{CN}\to {\mathrm{ET}H}^{•}+{\mathrm{CH}}_{2}{\mathrm{CN}}^{-}$$

2a $${\mathrm{ET}}^{•-}+{\mathrm{ET}H}^{•}⇄\mathrm{ET}+{\mathrm{ET}H}^{-}$$

2b $${\mathrm{ET}H}^{•}+X\to \mathrm{ET}+{\mathrm{XH}}^{•}$$

In step ([Disp-formula eq1]), the radical anion deprotonates the solvent, generating a neutral radical. In the second, step ([Disp-formula eq2a]), the ET^•–^ radical anion reduces the neutral radical or, step ([Disp-formula eq2b]), it undergoes a hydrogen abstraction by, for example, the solvent, which then decomposes. Alternatively, the reaction could be initiated by a disproportion reaction of the ET^•–^ radical anion [step ([Disp-formula eq3])], followed by deprotonation of acetonitrile by the ET^2–^ dianion [step ([Disp-formula eq4])]:

3 $${\mathrm{ET}}^{•-}+{\mathrm{ET}}^{•-}⇄\mathrm{ET}+{\mathrm{ET}}^{2-}$$

4 $${\mathrm{ET}}^{2-}+{\mathrm{CH}}_{3}\mathrm{CN}\to {\mathrm{ET}H}^{-}+{\mathrm{CH}}_{2}{\mathrm{CN}}^{-}$$

Based on the ca. 700 mV difference between the first and second reduction potentials of ET (Figure S9), the equilibrium in step ([Disp-formula eq3]) is far to the left at room temperature (ln *K* ≈ 28), but the irreversibility of step ([Disp-formula eq4]) will increase the formation of neutral ET. Both step ([Disp-formula eq1]) followed by step (2) and step ([Disp-formula eq3]) followed by step ([Disp-formula eq4]) produce CH_2_CN^–^ as a nucleophile and neutral ET.

The deprotonation of acetonitrile by ET^•–^ suggests that the stability of ET^•–^ can be affected by the choice of solvent. Figure S10 compares the CV curve of ET in acetonitrile with those of ET in propylene carbonate, dimethylformamide, dimethyl sulfoxide, and dimethoxyethane. After 10 cycles, the retention of the redox wave is lowest in acetonitrile (98.9%) and dimethoxyethane (98.7%), followed by dimethylformamide (99.4%) and propylene carbonate (99.7%), while virtually no decrease is seen in dimethyl sulfoxide (100%). This confirms the role of the solvent in the stability of ET^•–^. It suggests that a change to dimethyl sulfoxide is a useful strategy to create a more stable reduced state, but the 6-fold higher viscosity of dimethyl sulfoxide (1.99 cP) compared to acetonitrile (0.33 cP) makes it much less attractive for flow cell cycling. Similar arguments apply to dimethylformamide (0.80 cP) and propylene carbonate (2.51 cP).

### 3.3 DFT Calculations

To further investigate the degradation reaction mechanism,^39^ DFT calculations were performed at the ωB97XD/6-31+G** level of theory.^40,41^ Implicit solvation effects were modeled using the polarizable continuum model, with acetonitrile as the solvent to replicate experimental conditions.^42^ Transition-state structures were confirmed via vibrational frequency analysis, ensuring reliable characterization of the reaction pathway. In the first step, we consider that the CH_2_CN^–^ nucleophile formed in step ([Disp-formula eq1]) or ([Disp-formula eq4]) attacks the nitrile group (−CN) of the neutral terephthalonitrile, forming a new C–C bond and yielding Intermediate 1 (I_1_, Figure 4b). Next, hydrogen from the CH_2_CN group migrates in a [1,3]-H shift to the nitrogen of the original nitrile group, forming Intermediate 2 (I_2_), which has a lower energy because of the increased π conjugation. Finally, I_2_ abstracts a proton from the solvent to produce the degradation product that has been observed experimentally for ET.

The DFT calculations (Figure 4b) show that differences in the nature or position of the alkyl substituents, such as ethyl groups in ET (compare R_1_ = Et and R_2_ = H with R_1_ = H and R_2_ = Et) and DET (R_1_ = R_2_ = Et) and *tert*-butyl groups in DTBT (R_1_ = R_2_ = *t*-Bu), affect the activation free energy and that steric hindrance reduces the accessibility of the nitrile group for nucleophilic attack by CH_2_CN^–^. Specifically, the transition-state energy rises from 0.709 and 0.715 eV for the two ET isomers to 0.742 eV for DET and to 0.764 eV for DTBT, implying that bulkier substituents raise the energy barrier, making the degradation via nucleophilic attack less favorable.

For ET, two different regioisomers can be formed, depending on which of the two nitrile groups is attacked. Figure 4b shows that the transition-state energy for nucleophilic attack is only 0.006 eV higher when the nitrile group ortho to the ethyl group reacts (0.715 eV for R_1_ = H and R_2_ = Et) than the nitrile group that is positioned meta (0.709 eV for R_1_ = Et and R_2_ = H). The calculated difference in activation energies (0.006 eV) translates into an expected 1:1.3 distribution of the two regioisomers at room temperature, whereas experimentally only one regioisomer is formed, both in the degradation of ET^•–^ and in the reaction of ET with CH_3_CN and *t*-BuOK as a base.

The free energy profiles (Figure 4b) show that the intermediates formed during the degradation process are less stable in the presence of bulkier alkyl groups. Specifically, the Δ*G* values of I_1_ are 0.479 eV for DTBT, 0.442 eV for DET, 0.337 eV for ET (R_1_ = Et and R_2_ = H), and 0.436 eV for ET (R_1_ = H and R_2_ = Et). Hence, the barrier for the reverse reaction is higher when I_1_ is sterically less congested. This 0.1 eV difference pushes the equilibrium for ET more (by a factor of ca. 40) toward degradation when R_1_ = Et and R_2_ = H than when R_1_ = H and R_2_ = Et, which rationalizes why only one regioisomer is found experimentally. Similarly, I_2_ in DET (−0.589 eV) and DTBT (−0.508 eV) are less stable than that in ET (−0.602 eV). This trend continues through the isomerization and protonation steps, further confirming that steric hindrance plays a crucial role in slowing or blocking degradation via the nucleophilic attack of CH_2_CN^–^. The free energy profiles of the final product formation follow a similar trend. The Δ*G* values for adducts of CH_3_CN with DET (−1.789 eV), DTBT (−1.817 eV), and ET (−1.836 eV, for addition to the −CN group ortho to the ethyl group) indicate that the products formed in these systems gain less free energy than ET when addition occurs at the nitrile group that is meta to the ethyl group (−1.923 eV). These findings indicate that steric effects not only impact the transition-state and intermediate stability but also reduce the stability of the final degradation products. This aligns with experimental observations, where DET and DTBT were shown to exhibit slower degradation under the same conditions as those for ET (Figure 3). The increased transition-state energies and intermediate instability caused by bulky substituents support the hypothesis that steric hindrance mitigates the degradation reaction.

### 3.4 H-Cell and Redox-Flow Batteries with DET

Due to the limited stability of ET in the reduced state, we first continued with DET and evaluated its performance for redox-flow batteries. In combination with DBBTFB as a catholyte, DET gives a high cell potential of 3.22 V (Figure 5a). To assess the stability under charging and discharging conditions, a mixed solution of DET and DBBTFB was used in an H-cell. During 500 h of cycling, covering 406 cycles and two 3-day periods of keeping the battery in the charged state, the capacity dropped to approximately 41% of its initial value, implying an average capacity retention of 99.8% h^–1^ and 99.8% cycle^–1^ (Figure 5b). During these 500 h, the average Coulombic efficiency (CE) was 98%. CV measurements of the mixed solution after 500 h showed a retention of both the anolyte and catholyte of 43%, consistent with the drop in capacity (Figure S11a).

**Figure 5:**
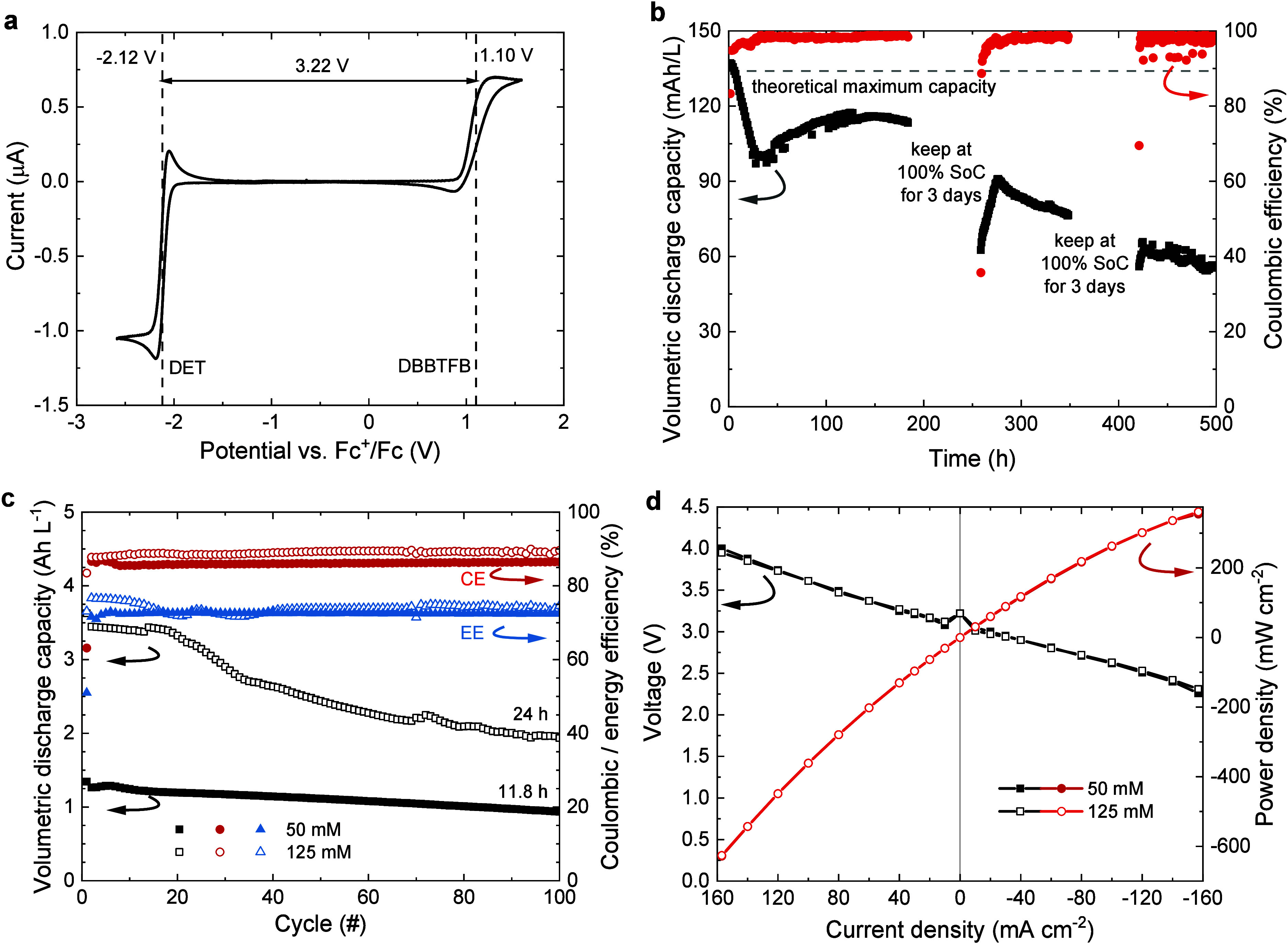
Characterization of H-cell and redox-flow batteries of DET and DBBTFB. (a) Microelectrode CV of a mixed solution of DET (50 mM) and DBBTFB (50 mM) in acetonitrile with TBAPF_6_ (300 mM). (b) Volumetric discharge capacity and CE vs time for 500 h and 406 cycles of a solution of DET (5 mM) with DBBTFB (5 mM) in acetonitrile with TBAPF_6_ (500 mM) using an H-cell. After 162 and 272 cycles, 3-day periods were included where the battery was prepared in a fully charged state and left untouched. (c) Volumetric discharge capacity, CE, and EE vs cycle number for a redox-flow battery consisting of a DET + DBBTFB solution in acetonitrile at 50 or 125 mM with TBAPF_6_ (300 mM). Charging was performed at a constant current of 40 mA cm^–2^, with a voltaic cutoff at 3.8 V. Discharging was performed at a constant current of −40 mA cm^–2^ with a voltaic cutoff at 2 V. (d) Voltage and power density vs current density during polarization at 0% SoC and depolarization at 100% SoC for the redox-flow batteries at 50 and 125 mM in acetonitrile with TBAPF_6_ (300 mM).

The capacity drop after pausing for 3 days at 100% SoC seen in Figure 5b is tentatively explained by the partial reoxidation of DET^•–^ to DET, similar to what is seen in Figure 3b. This causes a difference in the SoC values of the anolyte and catholyte compartments. As a result, after discharge, the catholyte compartment is not at 0% SoC, which limits the charging in the next cycle. The recovery of the volumetric capacity in subsequent cycles can be rationalized by the crossover of charges and neutral species that restores a balanced SoC.

Next, mixed anolyte–catholyte redox-flow batteries were cycled at a 50 mM concentration of each component in acetonitrile containing TBAPF_6_ (300 mM) (Figure 5c). A Daramic 175 porous separator was used because ion-exchange membranes would swell in acetonitrile, which results in a loss of selectivity while having a higher resistance.^22^ Under these conditions, the initial volumetric capacity of the redox-flow battery based on DET + DBBTFB is 1.27 Ah L^–1^, and it drops to 74% of that value during the first 100 cycles, corresponding to close to 12 h of operation. This implies average retentions of 99.6% cycle^–1^ and 97.0% h^–1^. After cycling, about 10% of volume transfer had occurred from the anolyte to the catholyte reservoir. The CE and EE remain fairly constant at values of 86% and 72% during cycling (Figure 5c). The DET + DBBTFB system was also tested for 100 cycles (24 h) at a higher concentration of 125 mM in acetonitrile with TBAPF_6_ (300 mM). This concentration corresponds to the solubility limit of the DBBTFB catholyte. At higher concentration, the volumetric capacity dropped to 56% from the initial value 3.35 Ah L^–1^ with an average volumetric capacity retention of 99.4% cycle^–1^ and 97.6% h^–1^ (Figure 5c). At 125 mM, the CE (89%) and EE (74%) are similar to the values at 50 mM.

Postcycling analysis of the anolyte reservoir with microelectrode CV shows a retention of the DET concentration of ca. 74% at 50 mM and 58% at 125 mM after 100 cycles, as inferred from the cathodic peak currents at −2.1 V, while in the catholyte reservoir, the DET concentration dropped to 74% for both concentrations tested (Figure S11c,e). The retention of DET after cycling is similar to the retention of the volumetric capacity (Figure 5c). This implies that, in this experiment, the retention is primarily related to the stability of DET.

Polarization and depolarization measurements of redox-flow batteries containing mixed solutions of DET with DBBTFB were investigated at 0% and 100% SoC at concentrations of 50 and 125 mM in acetonitrile containing TBAPF_6_ (300 mM) (Figure 5d). The maximum power densities are very similar and reached 355 and 361 mW cm^–2^ at 50 and 125 mM, respectively, at the maximum current density of 157 mA cm^–2^ that can be achieved by the potentiostat. With that, the DET + DBBTFB redox-flow battery outperforms previously reported nonaqueous flow batteries (Table S1),^22,25,43–47^ where 336 mW cm^–2^ at about 160 mA cm^–2^ is the highest reported to date.^48^ It is also in the range of the best all-organic aqueous redox-flow batteries.^49–51^ Extrapolation of the data suggests a maximum power density of about 470 mW cm^–2^ at a current density of 300 mA for the DET + DBBTFB system.

### 3.5 Redox-Flow Batteries with DTBT

For the redox-flow battery of DTBT + DBBTFB, the cell potential is 3.15 V (Figure 6a). This is slightly less than that for DET + DBBTFB due to the 70-mV-less negative reduction potential of DTBT compared to DET. The redox-flow battery had a somewhat lower initial capacity than expected but reached an average retention of 99.8% cycle^–1^ and 98.1% h^–1^ with CE and EE of 93% and 77% during the first 100 cycles (Figures 6b and S12). After cycling, about 12.5% of anolyte solution had transferred to the catholyte reservoir. Postcycling analysis with CV showed a retention of the concentration of DTBT of ca. 83%, as inferred from the cathodic peak current at −2.1 V (Figure S13a), which matches to the retention inferred from the redox-flow battery cycling in Figure 6b (84%).

**Figure 6:**
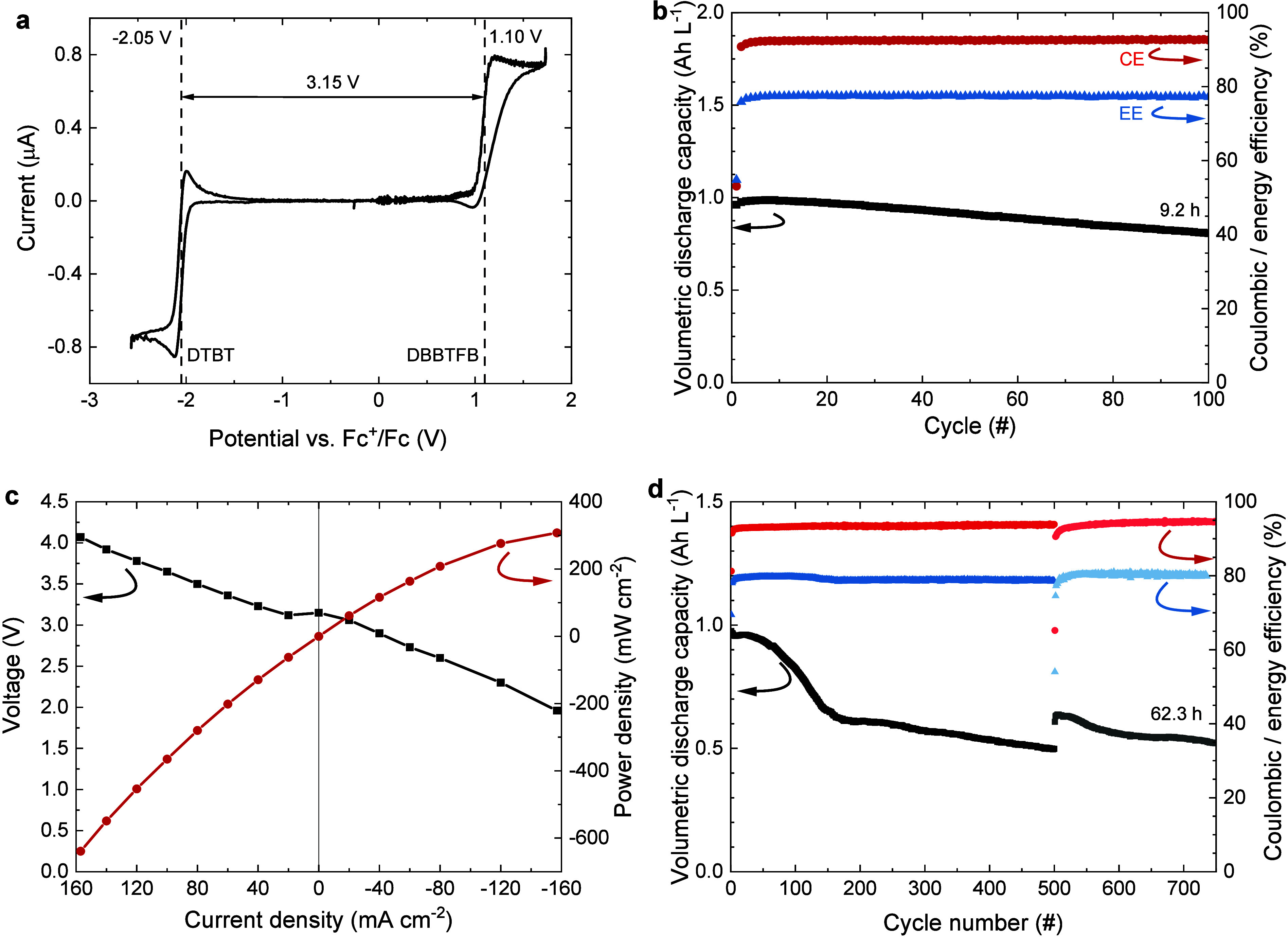
Characterization of redox-flow batteries DTBT with DBBTFB and DBBB. (a) Microelectrode CV of a mixed solution of DTBT (50 mM) and DBBTFB (50 mM) in acetonitrile with TBAPF_6_ (300 mM). (b) Volumetric discharge capacity, CE, and EE vs cycle number of a redox-flow battery consisting of a mixed solution of DTBT (50 mM) with DBBTFB (50 mM) in acetonitrile with TBAPF_6_ (300 mM). Charging was performed at a constant current of 40 mA cm^–2^, with a voltaic cutoff at 3.6 V. Discharging was performed at a constant current of −40 mA cm^–2^ with a voltaic cutoff at 2 V. (c) Voltage and power density vs current density during polarization at 0% SoC and depolarization at 100% SoC for the redox-flow battery of a mixed solution of DTBT (50 mM) with DBBTFB (50 mM) in acetonitrile with TBAPF_6_ (300 mM). (d) Volumetric capacity, CE, and EE vs cycle number of redox-flow battery cycling of a mixed solution of DTBT (50 mM) with DBBB (55 mM) in acetonitrile with TBAPF_6_ (200 mM) over an operation time of 62.3 h after 750 cycles. The polarity was reversed after 500 cycles. Charging was performed at a constant current of 30 mA cm^–2^, with a voltaic cutoff at 3.1 V. Discharging was performed at a constant current of −30 mA cm^–2^ with a short voltaic hold at 1 V (threshold 6 mA cm^–2^).

Postcycling analysis via ^1^H NMR of the DTBT + DBBTFB flow battery solution revealed that no major new products are formed after 100 cycles (Figure S14a). However, upon zooming in, the spectrum reveals new low-intensity signals in the aromatic range from 7.48 to 8.18 ppm (Figure S14b), hinting toward a slow degradation of the redox-active materials. These new signals are not compatible with the expected pattern for an adduct of DTBT and acetonitrile [i.e., 4-(1-amino-2-cyanovinyl)-2,5-di-*tert*-butylbenzonitrile]. This confirms that the two bulky *tert*-butyl groups are effective in reducing the level of adduct formation observed for ET. Instead, the signals between 7.48 and 7.74 ppm are tentatively assigned to 2,5-di-*tert*-butylbenzonitrile,^52^ probably formed from DTBT^•–^ via loss of CN^**–**^ and abstraction of a hydrogen atom from the solvent. The peak at 8.18 ppm is possibly due to 3,6-di-*tert*-butylbenzene-1,2,4-tricarbonitrile. These degradation products could not be isolated, and, hence, the assignments could not be confirmed.

Polarization and depolarization measurements of the redox-flow batteries containing mixed solutions of DTBT with DBBTFB were investigated at 0% and 100% SoC at a concentration of 50 mM in acetonitrile containing TBAPF_6_ (300 mM) (Figure 6c). The maximum power density reached is 307 mW cm^–2^ for DTBT + DBBTFB at the maximum current density of 157 mA cm^–2^.

An improved stability was achieved by replacing DBBTFB with DBBB. The lower oxidation potential of DBBB reduces the battery voltage to 2.76 V. The volumetric capacity of the mixed DTBT + DBBB redox-flow battery dropped to 54% of the initial value over an operation time of 62.3 h (750 cycles), resulting in average retention of 99.92% cycle^–1^ or 99.0% h^–1^ and CE and EE of 93% and 80% (Figure 6d). The higher stability of the system could be due to DBBB being more stable than DBBTFB or the lower current that was applied. After an initial drop between cycles 50 and 150, the volumetric capacity remained fairly constant. The fast capacity drop within the first 150 cycles is mainly due to volume transfer between reservoirs, which can occur as a result of differences in hydraulic pressure at either side of the separator.^53,54^ After 150 cycles, about 1.25 mL out of a total of 5 mL had transferred from the anolyte reservoir to the catholyte reservoir, including active compounds and electrolyte salt. Analyzing both reservoirs after 500 cycles with microelectrode CV (Figure S13b) revealed that at least 75% of molecule DTBT remained in the anolyte reservoir and 80% in the catholyte reservoir. Based on these numbers, the capacity retention would be between 99.94% and 99.96% cycle^–1^. After 500 cycles and measurement of the CV, the polarity of the battery was reversed, and another 250 cycles were performed, which show a trend similar to that seen in cycles 150–500, confirming the good cyclability and the opportunity for polarity inversion to mitigate the consequences of volume transfer. After a total of 750 cycles, including one polarity reversal, the volumetric capacity dropped to about 53.5% of the initial value, implying a capacity retention of 99.92% cycle^–1^. The volumes of both reservoirs were similar after 750 cycles.

## 4 Conclusions

The alkyl-substituted terephthalonitriles are easy-to-synthesize anolytes for nonaqueous redox-flow batteries. Their deep reduction potentials enabled redox-flow batteries in acetonitrile to achieve a high cell voltage of 3.22 V when using DET as the anolyte and DBBTFB as the catholyte. An H-cell battery showed a stability of 99.8% cycle^–1^ for more than 500 h and 406 cycles at a concentration of 5 mM. In a redox-flow battery, the volumetric capacity retention was up to 99.6% cycle^–1^ at 50 mM and 99.4% cycle^–1^ at 125 mM. The degradation is primarily due to loss of the redox-active compound. For ET, we identified a degradation reaction in which the ET^•–^ radical anion abstracts a proton from acetonitrile, which subsequently reacts with neutral ET in an overall addition reaction. Experiments and DFT calculations revealed that the stability of the terephthalonitrile radical anion increases by introducing (bulky) alkyl side chains, which delay the addition reaction via steric hindrance. Consistently, introducing *tert*-butyl groups in DTBT increased the capacity retention to 99.8% cycle^–1^ for a 3.15-V redox-flow battery with DBBTFB. For this combination, postcycling analysis revealed the formation of another side product, likely formed via loss of CN^–^ from the reduced anolyte, followed by abstraction of a hydrogen atom from the solvent. Replacing DBBTFB by DBBB as the catholyte resulted in a 2.76-V flow battery with an average volumetric capacity retention of 99.9% cycle^–1^ for 750 cycles and EE of 80% cycle^–1^. This is higher than previously published redox-flow batteries, achieving similar cell voltage and concentrations (Table S1).

Together, these results demonstrate that alkyl-substituted terephthalonitriles represent an interesting class of anolyte materials for organic redox-flow batteries. However, for future practical applications, both solubility and stability must be increased. Polar side chains can enhance the solubility. To enhance the stability above 99.9% cycle^–1^, it is necessary to reduce the reactivity of the terephthalonitrile radical anions toward the solvent and toward the loss of cyanide ions. Further derivatization with electron-withdrawing substituents can likely accomplish this but will reduce the battery voltage.

## Supplementary Material



## Notes

Synthesis and characterization of anolytes and catholytes (^1^H NMR, ^13^C NMR, and mass spectrometric analysis), additional table and figures, and NMR spectra (PDF)
