# Supplementary material for: Supra-3‑V Nonaqueous Redox-Flow Batteries Based on Simple Terephthalonitrile Anolytes
Source: ACS Appl Energy Mater. 2025 Oct 6;8(20):15124–33. doi: 10.1021/acsaem.5c01949 (PMC12569969; doi:10.1021/acsaem.5c01949)
Supplement: Supplementary file 1 [file ae5c01949_si_001.pdf]

Supporting Information

Supra-3-Volt Nonaqueous Redox Flow Batteries Based on Simple Terephthalonitrile Anolytes

Nicolas Daub,[1] Xiaotong Zhang,[2] Nico J. L. van Rijswijk,[1] Piotr de Silva,[2] and René A. J. Janssen[1,3]*

[1] Molecular Materials and Nanosystems & Institute for Complex Molecular Systems, Eindhoven University of Technology, P.O. Box 513, 5600 MB Eindhoven, The Netherlands, E-mail: r.a.j.janssen@tue.nl

[2] Department of Energy Conversion and Storage, Technical University of Denmark, Anker Engelunds Vej 301, 2800 Kongens Lyngby, Copenhagen, Denmark, E-mail: pdes@dtu.dk

[3] Dutch Institute for Fundamental Energy Research, De Zaale 20, 5612 AJ Eindhoven, The Netherlands

Table-of-contents

Synthetic procedures and characterization S2

Table S1 S4

Figures S1–S18: S6

Supplementary references S24

Synthetic procedures and characterization

Dry solvents were purchased from Thermo Fisher Scientific and used as received. 2,5-Di-*tert*-butylhydroquinone (Sigma-Aldrich/Merck), 1,4-dibromo-2,5-diethylbenzene (TCI), 1,4-di-*tert*-butylbenzene (Acros), 2,2,2-trifluoroethyl trifluoromethanesulfonate (TCI), and cesium carbonate (abcr GmbH), and were obtained from commercial sources. If not stated differently, all chemicals were used without further purification. All reactions were performed under nitrogen atmosphere. NMR spectra were obtained on a Bruker 400 MHz spectrometer. ^{1}H and ^{13}C chemical shifts are reported in parts per million (ppm) relative to TMS, with the residual solvent peak used as an internal reference.

2,5-Diethylterephthalonitrile (DET)

DET was prepared following the procedure of Sugamata et al.[S1] 1,4-Dibromo-2,5-di-ethylbenzene (0.30 g, 1.03 mmol) was dissolved in anhydrous *N,N*-dimethylformamide (DMF) (0.2 M), copper cyanide (0.20 g, 2.26 mmol) was added, and the mixture was stirred under reflux for 3 h. After cooling, the mixture was poured into 10% aqueous ammonia and extracted three times with dichloromethane (DCM). The combined organic phase was washed twice with demineralized water, dried over $MgSO_4$, and concentrated under vacuo. The crude mixture was recrystallized from heptane to yield 169 mg of **DET** as white crystals (90%). ^{1}H NMR (400 MHz, Chloroform-*d*) δ 7.57 (s, 2H), 2.88 (q, J = 7.5 Hz, 4H), 1.31 (t, J = 7.5 Hz, 6H) (Figure S15). ^{13}C NMR (100 MHz, Chloroform-*d*) δ 146.14, 132.89, , 27.20, 14,78 (Figure S16). The ^{1}H NMR spectrum is in agreement with the literature.[S1,S2]

1,4-Dibromo-2,5-di-*tert*-butylbenzene

1,4-Di-*tert*-butylbenzene (3 g, 15.76 mmol) was dissolved in chloroform (0.5 M) and the solution cooled to 0 °C. Bromine (1.63 mL, 31.52 mmol) and iodine (0.40 g, 1.58 mmol) were added, and the mixture was stirred overnight. After pouring slowly into 20% aqueous sodium hydroxide the organic phase was separated and the aqueous phase was extracted twice with DCM. The combined organic phase was washed with demineralized water, dried over $MgSO_4$, and concentrated under vacuo. The crude mixture was recrystallized from heptane to yield 2.47 g of 1,4-dibromo-2,5-di-*tert*-butylbenzene as white crystals (45%). ^{1}H NMR (400 MHz, Chloroform-*d*) δ 7.58 (s, 2H), 1.47 (s, 18H). The ^{1}H NMR spectrum is in agreement with the literature.[S3]

2,5-Di-*tert*-butylterephthalonitrile (DTBT)

1,4-Dibromo-2,5-di-*tert*-butylbenzene (0.5 g, 1.44 mmol) was dissolved in anhydrous DMF (0.2 M), copper cyanide (0.32 g, 3.59 mmol) was added, and the mixture was stirred under reflux overnight. After cooling, the mixture was poured into 10% aqueous ammonia and extracted 3 times with DCM. The combined organic phase was washed with demineralized water, dried over $MgSO_4$, and concentrated under vacuo. The crude mixture was recrystallized from heptane to yield 155 mg of molecule **DTBT** as white crystals (45%). ^{1}H NMR (400 MHz, Chloroform-*d*) δ 7.74 (s, 2H), 1.52 (s, 18H) (Figure S17). ^{13}C NMR (100 MHz, Chloroform-*d*) δ 151.75, 133.54, 119.22, 114.89, 35.52, 30.01 (Figure S18). The ^{1}H and ^{13}C NMR spectra are in agreement with literature.[S4,S5]

1,4-Di-*tert*-butyl-2,5-bis(2,2,2-trifluoroethoxy)benzene (DBBTFB)

The synthesis is based on a procedure of Bheemireddy et al.[S6] 2,5-di-*tert*-butylhydroquinone (0.25 g, 1.12 mmol) was dissolved in anhydrous DMF, Cs_2CO_3 (1.83 g, 5.62 mmol) added and heated to 90 ºC. Dropwise addition of 2,2,2-trifluoroethyl trifluoromethanesulfonate (0.61 mL, 4.50 mmol) turned the orange solution into a bright yellow slurry (bright ocher after 1 h) which was stirred overnight. To the light brownish slurry, 50 mL of ethyl acetate were added, and the solution extracted with $NaHCO_3$ and brine. After drying over $MgSO_4$ and evaporation, 334 mg of a yellow solid were obtained. Recrystallization from 4 mL of ethanol/heptane (6:1 v/v) yielded 295 mg off white needles (68%). ^{1}H NMR (400 MHz, Chloroform-*d*) δ 6.77 (s, 2H), 4.36 (q, J = 8.0 Hz, 2H), 1.36 (s, 18H). ^{13}C NMR (100 MHz, Chloroform-*d*) δ 150.62, 137.55, 112.27, 65.29 (q), 34.76, 29.74. The ^{1}H and ^{13}C NMR spectra are in agreement with the literature.[S6]

Table S1. Comparison of performance parameters of anolytes.

Structure of anolyte	Structure of catholyte	$E_{1/2}$ vs. Fc^{+}/Fc (V)	Battery voltage [a] (V)	Decay per cycle	Energy efficiency at J	Ref.
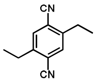	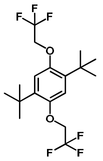	−2.12	3.22	0.32% (0.05 M)<br>0.56% (0.125 M)	72%<br>(40 mA cm^{-2})<br>74%<br>(40 mA cm^{-2})	
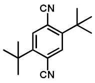	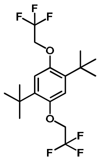	−2.05	3.15	0.17% (0.05 M)	77%<br>(40 mA cm^{-2})	This work
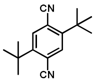	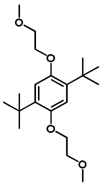	−2.05	2.76	0.08% (0.05 M)	80%<br>(30 mA cm^{-2})	
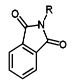	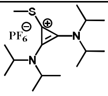	−1.87	3.18	1.8%[b] (0.05 M)	70%<br>(10 mA cm^{-2})	S7
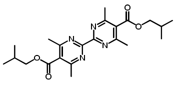	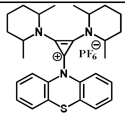	−1.88,<br>−2.05[c]	2.52,<br>3.00	0.07% (0.025 M)	Not given<br>(15 mA cm^{-2})	S8
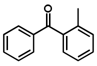	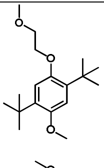	−2.34	2.97	0.42% (0.05 M)	70%<br>(7.5 mA cm^{-2})	S9
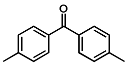	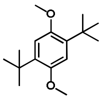	−2.34	2.97	0.96% (0.05 M)	34%<br>(1.0 mA cm^{-2})	S10
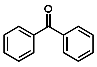	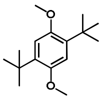	−2.28	2.95	0.54% (0.1 M)	44%<br>(1.0 mA cm^{-2})	S11
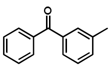	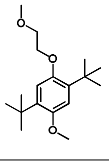	−2.27	2.89	– [d]	66%<br>(7.5 mA cm^{-2})	S12

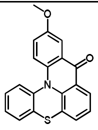	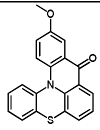	−2.13[e]	2.76	0.61 (0.025 M)	87% (10 mA cm^{-2})	S13
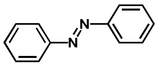	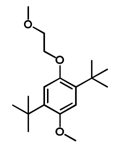	−1.76	2.5	≈0.7% (0.05 M) ≈0.9% (0.25 M)	70% (80 mA cm^{-2}) 58% (100 mA cm^{-2})	S14
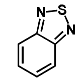	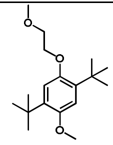	−1.67[e]	2.36	0.11% (0.1 M)	70% (40 mA cm^{-2})	S15
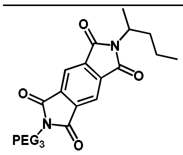	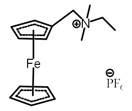	−1.26, −1.88[c]	1.51, 2.09	0.8% (0.5 M of e^-)	72% (40 mA cm^{-2})	S16
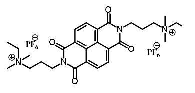	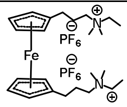	−0.82, −0.95[c]	0.75, 0.87	0.017% (0.2 M of e^-)	Not given	S17

[a] Measured values. [b]Decay of 0.35%/cycle for the catholyte according to CV, 1.8% for the anolyte (battery is limited by anolyte decaying). [c] The molecule exhibits two reversible consecutive single-reduction events. [d] Necessary data not provided. [e] Calculated by subtracting 85 mV from the potential vs. Ag^+/Ag.

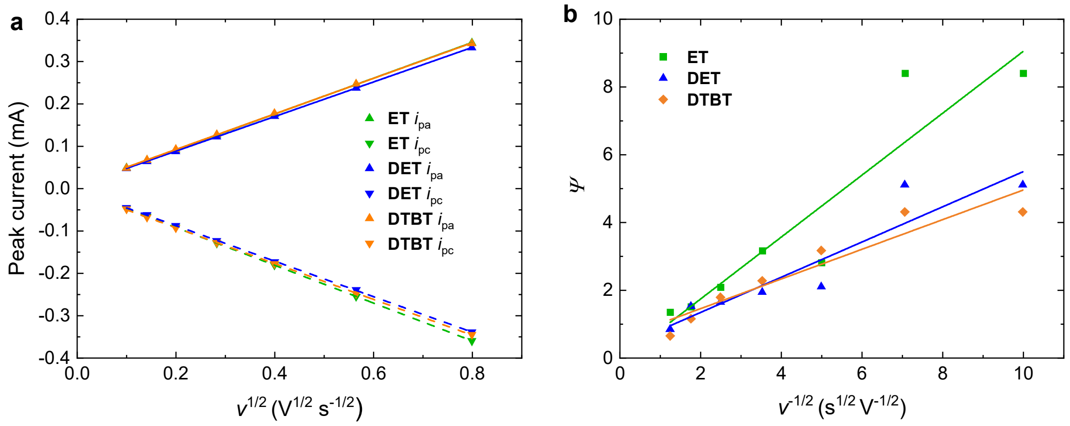

Figure S1. Determination of diffusion constants (D) and electron transfer constants (k_0). a) Peak currents (i_{pa} and i_{pa}) obtained from Figure 2 vs. square root of the scan speed ($v^{1/2}$). The anodic peak currents were used to determine the diffusion coefficients listed in Table 1 for the single-electron reduction of the compounds **ET, DET**, and **DTBT**. Lines are least-squares fits to the data. b) Plot of $\Psi = \gamma k_0 \big/ \sqrt{\pi n F v D / RT}$ vs. $v^{-1/2}$ where $\gamma = D/D'$ (the ratio of the diffusion constants for the reduction and re-oxidation), F the Faraday constant, R the gas constant, T the temperature and n the number of electrons transferred used to determine the electron transfer constants k_0 listed in Table 1 for the single-electron reduction of the compounds **ET, DET**, and **DTBT**. Lines are least-squares fits to the data.

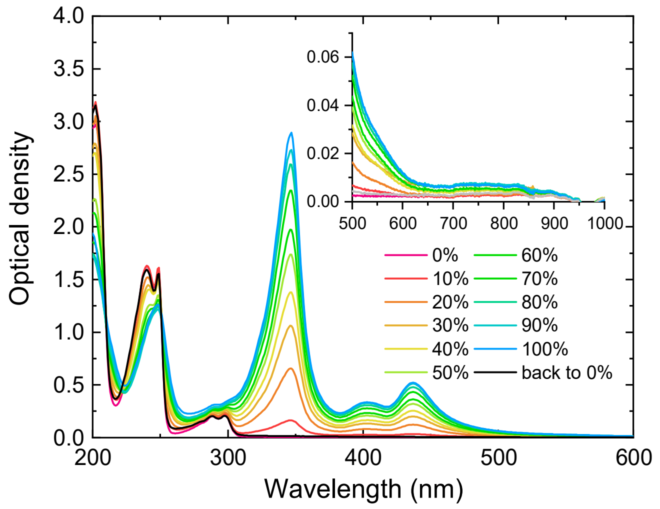

Figure S2. UV-vis-NIR absorption spectra of ET at different state of charge. Spectra were recorded for **ET** (50 mM) in acetonitrile with $TBAPF_6$ (200 mM) in a 0.2 mm cuvette.

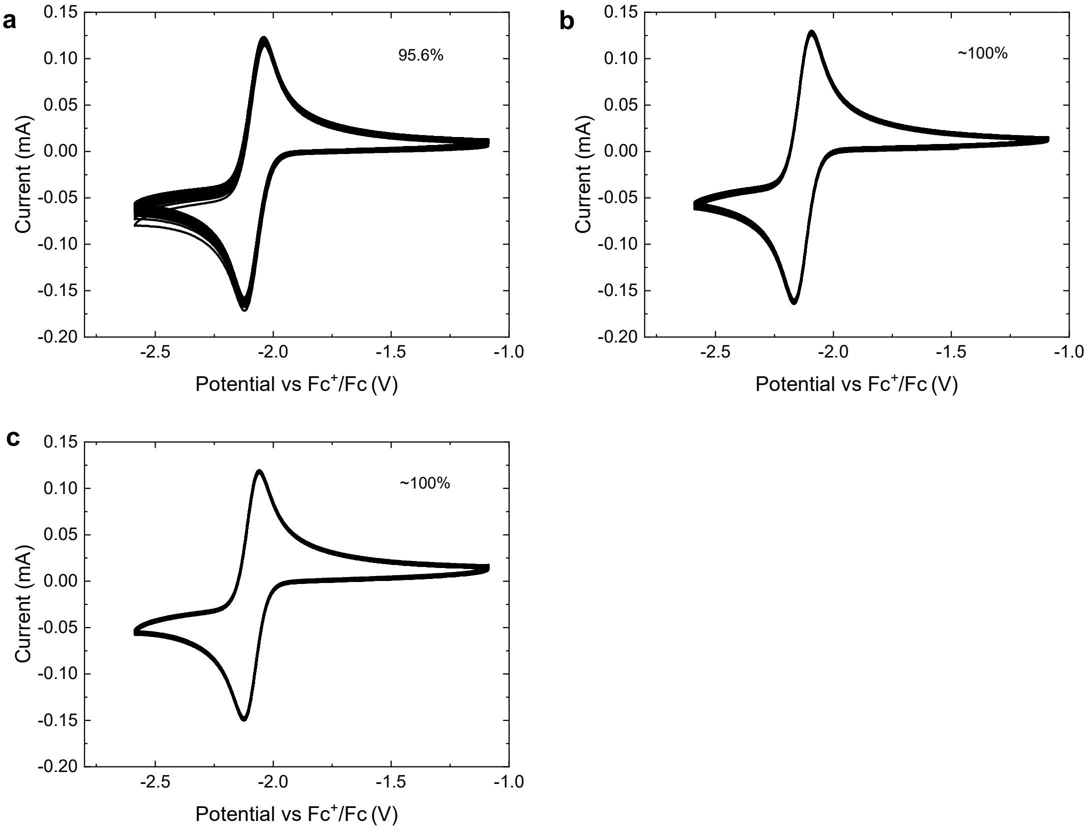

Figure S3. Cyclic voltammograms of 5 mM solutions of anolytes in acetonitrile containing $TBAPF_6$ (200 mM). a) **ET**. b) **DET**. c) **DTBT.** In each case 100 consecutive cycles are shown. The retention of the signal after 100 cycles is given in the panels.

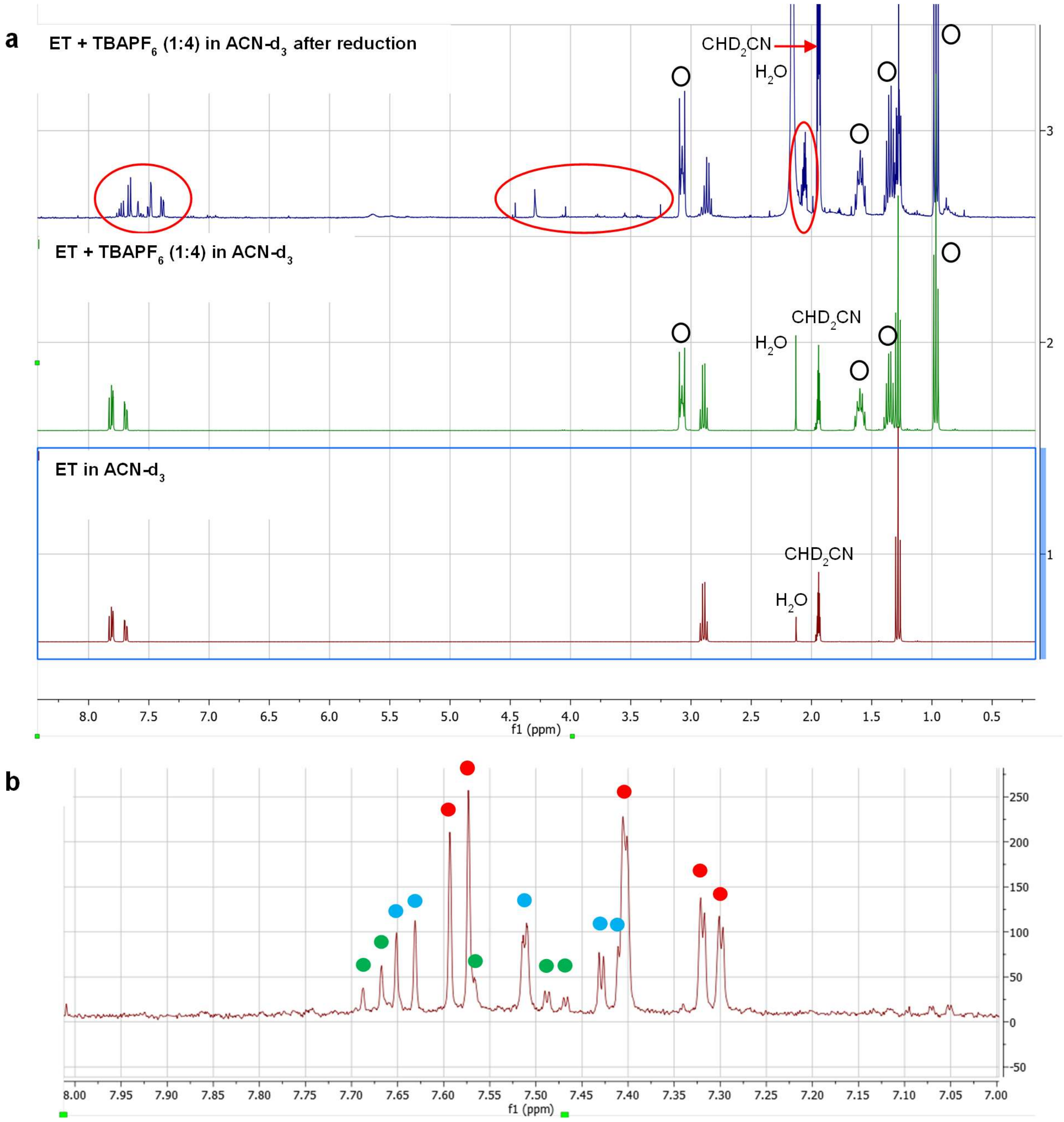

Figure S4. NMR spectra of ET after electrochemical reduction and storage. a) ^{1}H NMR spectra (CD_3CN) for **ET** (bottom), **ET** with $TBAPF_6$ in a 1:4 molar ratio before electrochemical reduction (middle) and after storing the reduced **ET** solution for 6 days (top). Most intense signals of degradation and side products are indicated with red ovals. Open circles correspond to TBA^+. Impurity signals of CHD_2CN and H_2O are indicated. b) Zoom-in of the aromatic region of the degraded sample. Colored dots identify three different ABC-coupled spin systems, each corresponding to a 1,2,4-subsituted benzene ring.

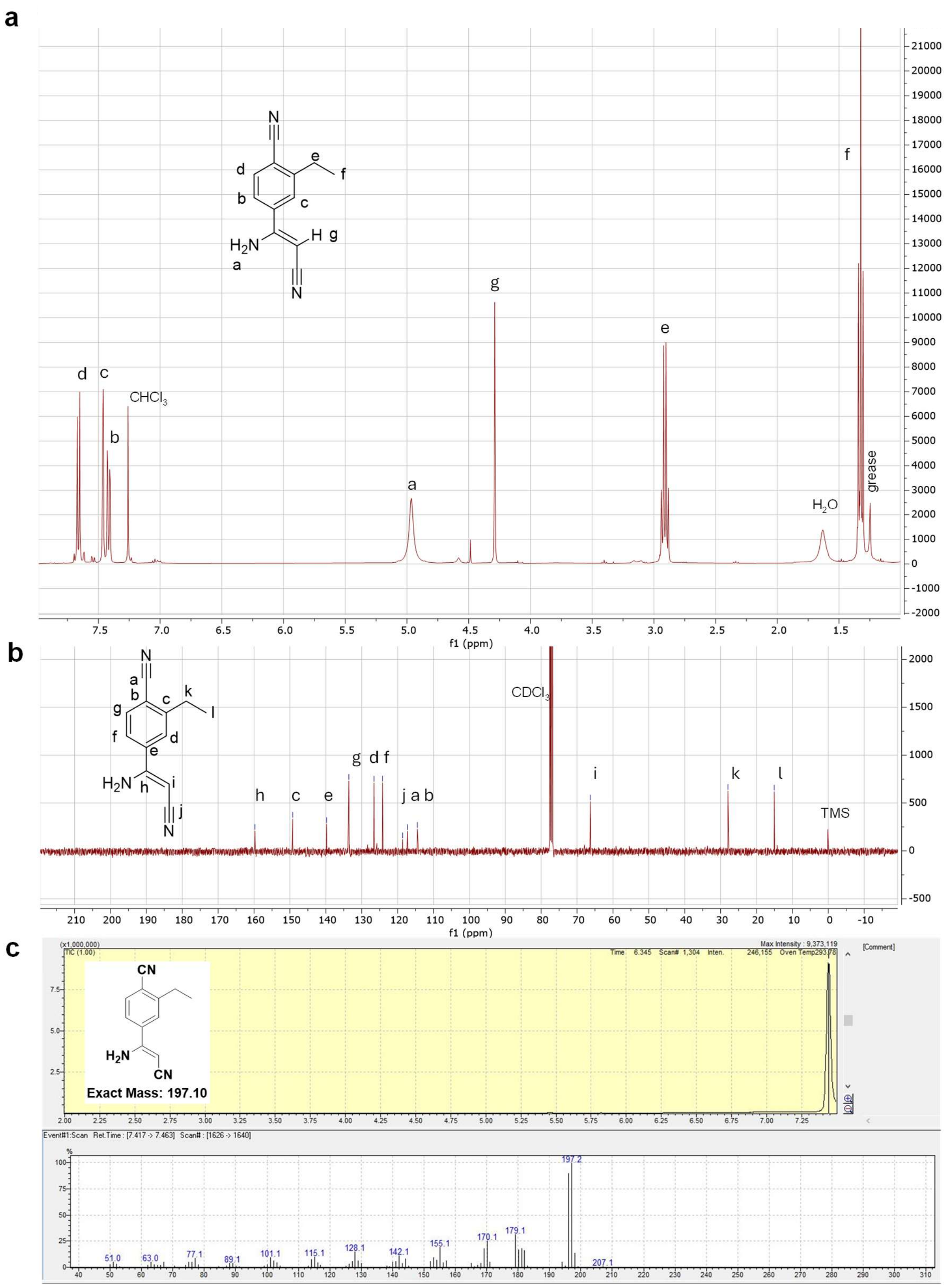

Figure S5. Analysis of the isolated degradation product (*Z*)-4-(1-amino-2-cyanovinyl)-2-ethylbenzonitrile. a) ^{1}H NMR spectrum recorded in $CDCl_3$. b) ^{13}C NMR spectrum recorded in $CDCl_3$. c) GC-MS.

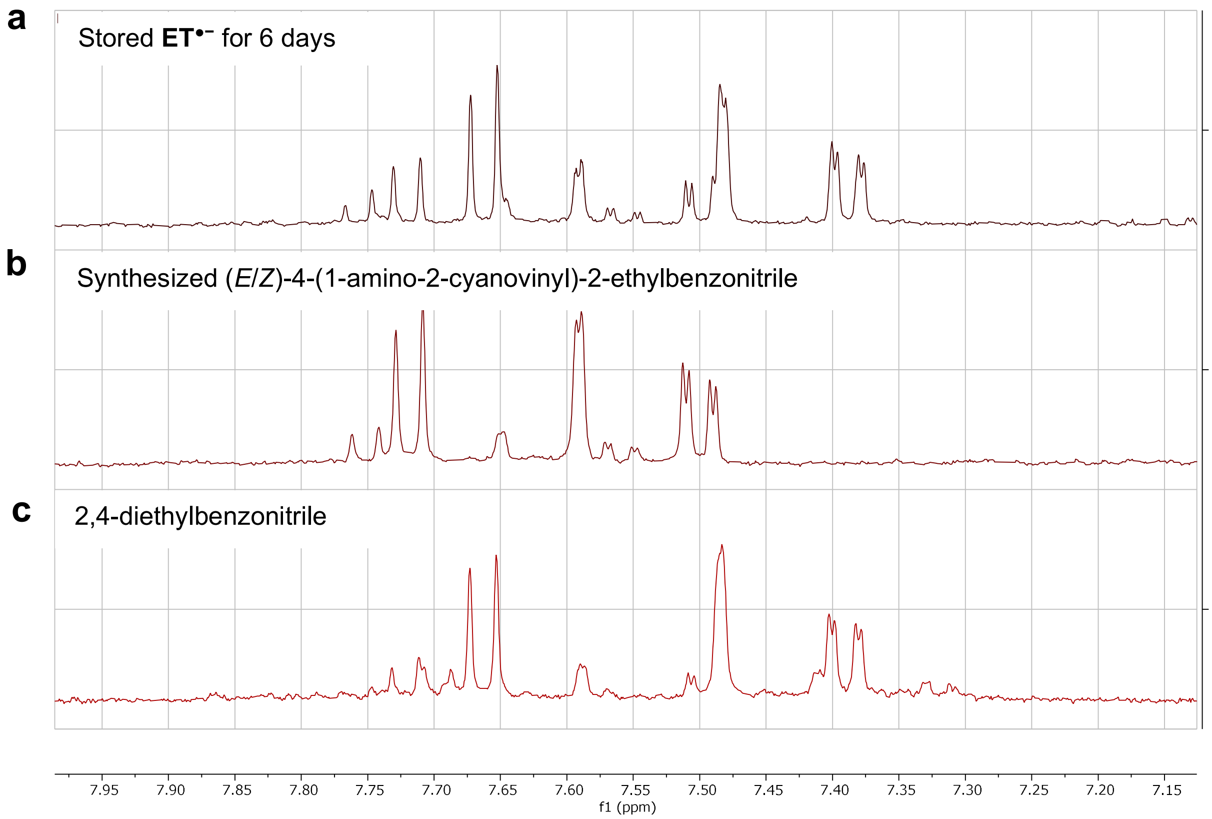

Figure S6. Comparison of the ^{1}H NMR spectra of the crude and purified products obtained after electrochemical reduction and storage of ET. a) The crude product obtained from the degraded sample of **ET**$^{•−}$ (Figure S4). b) (*E*/*Z*)-4-(1-amino-2-cyanovinyl)-2-ethylbenzonitrile synthesized from **ET** and CH_3CN in the presence of *t*-BuOK. c) The spectrum of (impure) 2,4-diethylcyanobenzene (Figure S7). The spectra shown in panels b) and c) identify the three main products found in the degraded sample of **ET**$^{•−}$ as 2,4-diethylcyanobenzene and (*E*/*Z*)-4-(1-amino-2-cyanovinyl)-2-ethylbenzonitrile.

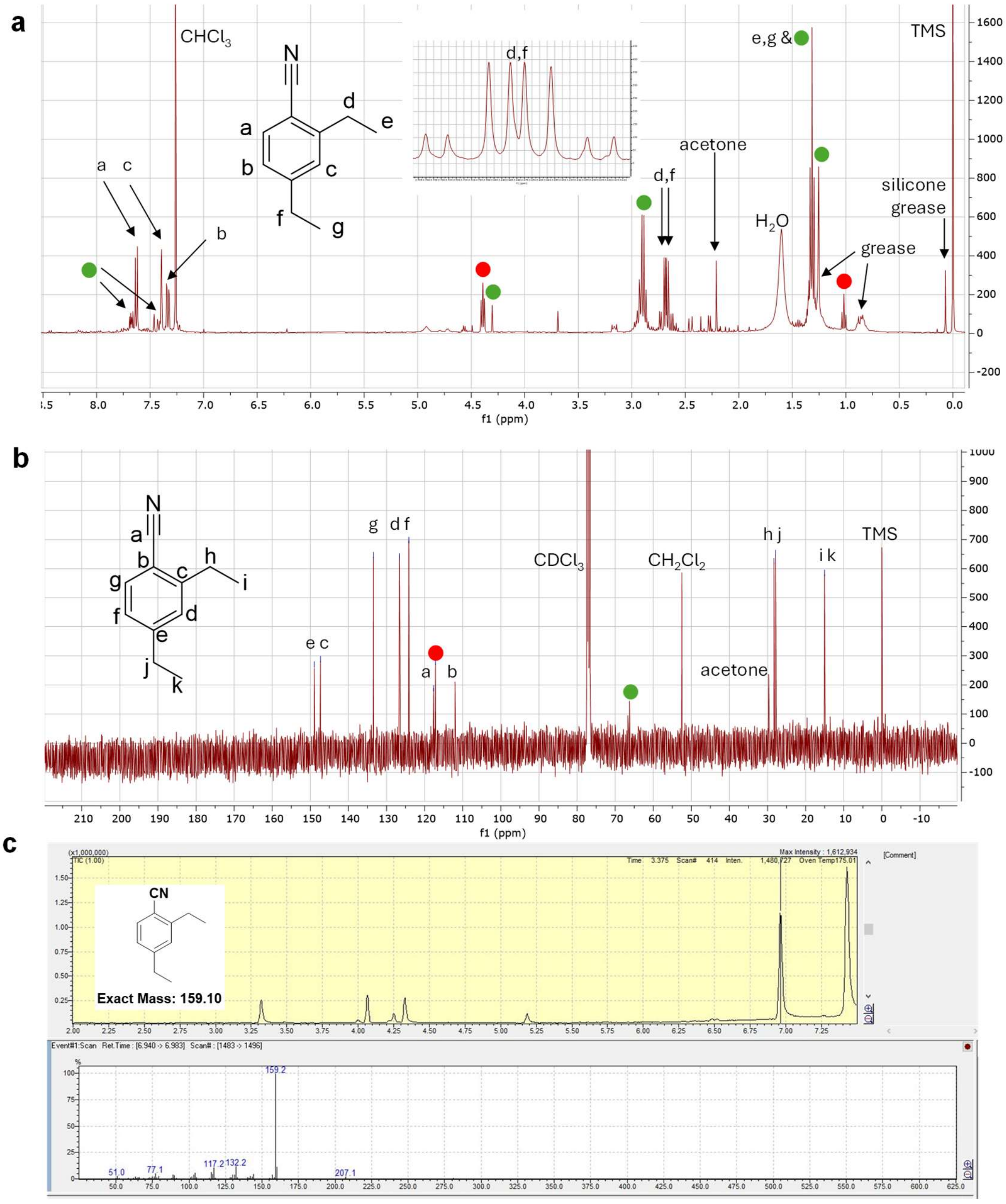

Figure S7. Analysis of the isolated degradation product 2,4-diethylbenzonitrile a) ^{1}H NMR spectrum spectra recorded in $CDCl_3$. b) ^{13}C NMR spectrum recorded in $CDCl_3$. **c,** GC-MS of the isolated degradation product. Peaks of residual solvents and TMS are indicated. The inset in the top panel shows a zoom-in on the two quartets expected for the methylene protons (d and f). The green dots are assigned to residual (*E*/*Z*)-4-(1-amino-2-cyanovinyl)-2-ethylbenzonitrile. The red dots and unlabeled peaks are unidentified impurities.

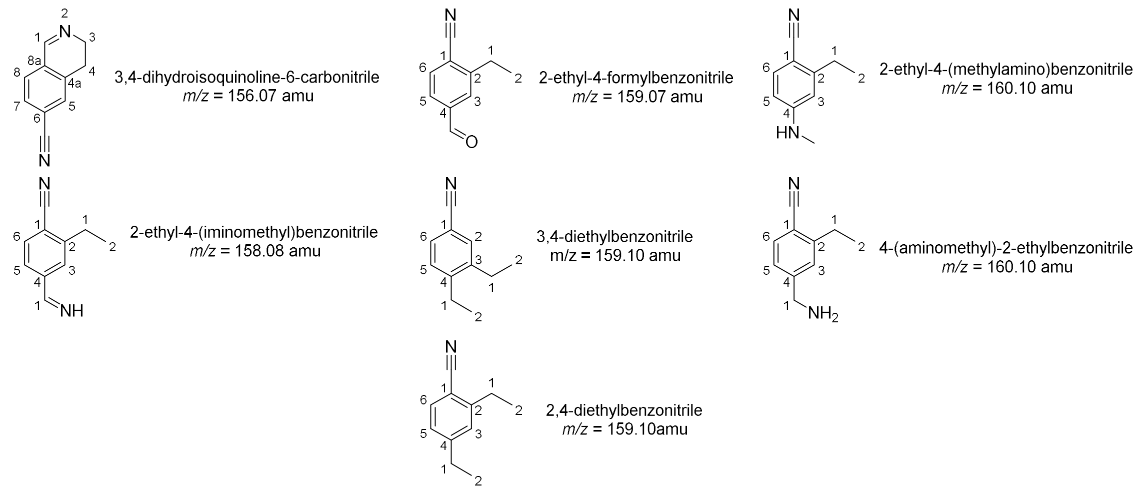

Figure S8. Structures of products considered with *m/z* in the range from 156 to 160 amu to explain the *m/z* = 159 amu product.

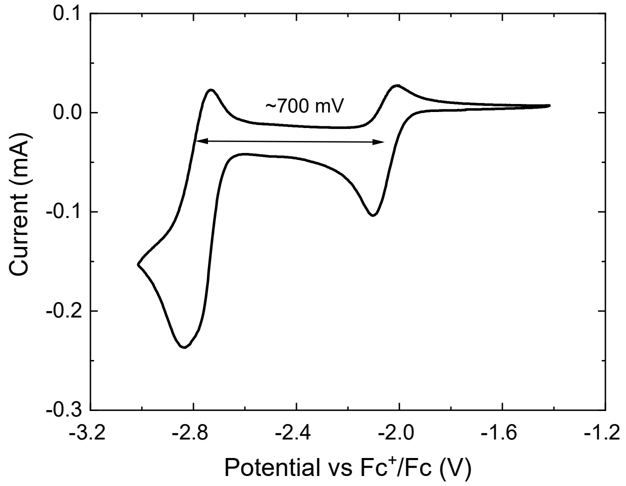

Figure S9. Cyclic voltammogram of ET dissolved in acetonitrile (5 mM) containing $TBAPF_6$ (200 mM) recorded at a scan rate of 100 mV s^{-1}.

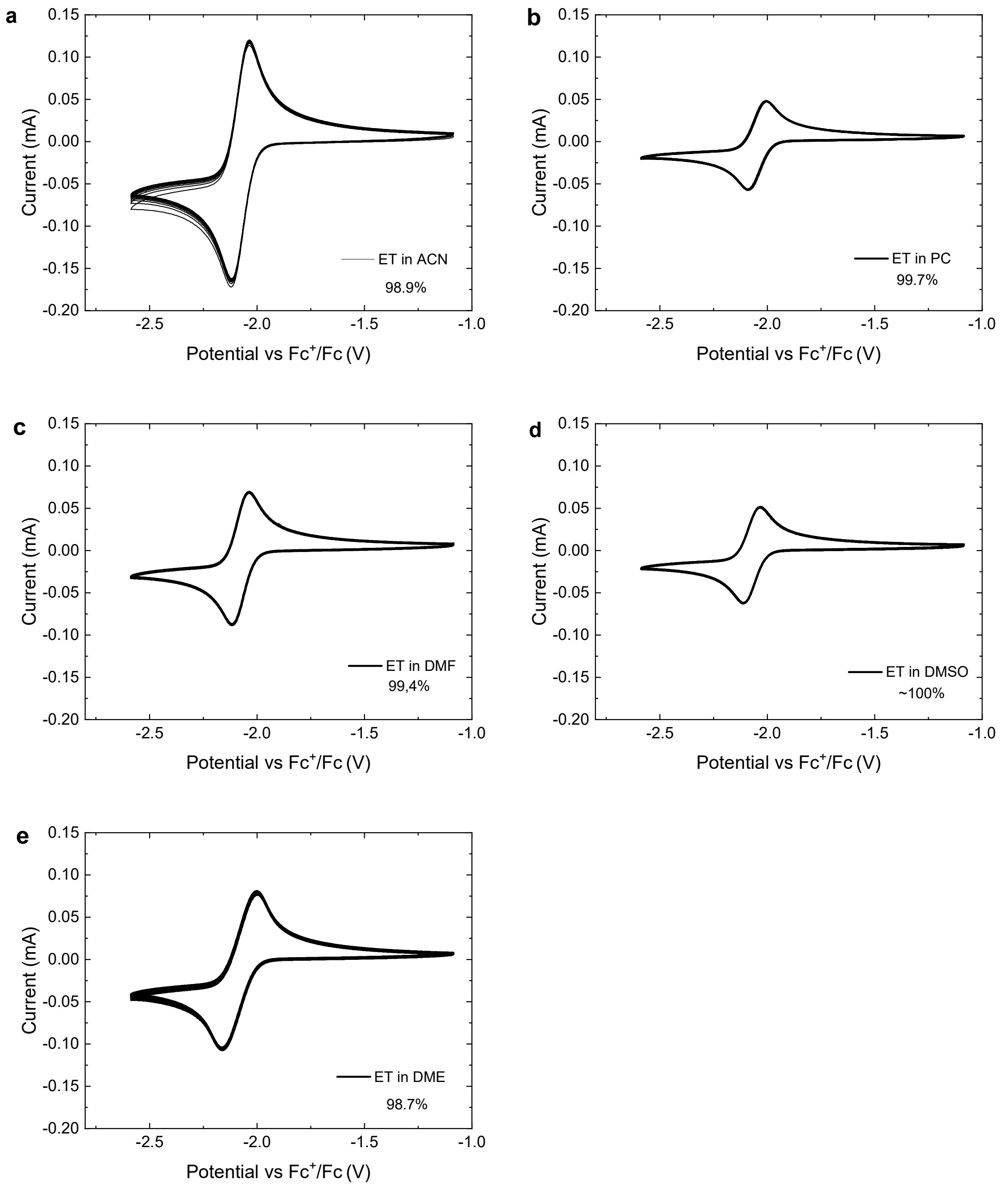

Figure S10. CVs of **ET** (5 mM) in different solvents with $TBAPF_6$ (200 mM) over 10 cycles. a) Acetonitrile. b) Propylene carbonate. c) Dimethylformamide. d) Dimethyl sulfoxide. e) Dimethoxyethane.

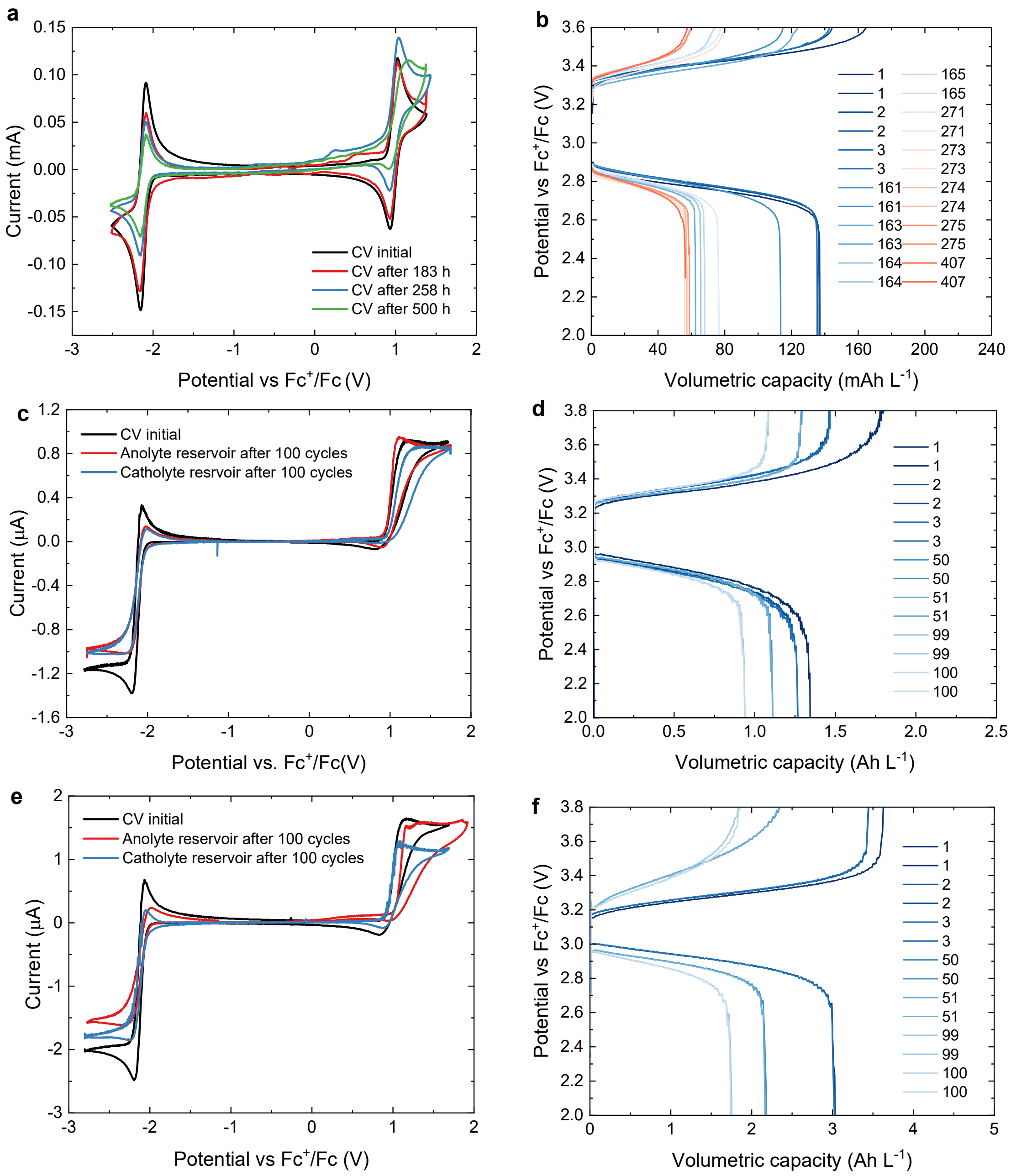

Figure S11. a) CV of the anolyte compartment of the H-cell cycling shown in Figure 5b before and after 183, 258, and 500 h of cycling. b) Charge-discharge curves of the cycling shown in Figure 5b. c) Microelectrode CVs of anolyte and catholyte reservoirs of the redox flow batteries shown in Figure 5c before and after 100 cycles at 50 mM d) Charge-discharge curves of the 50 mM cycling shown in Figure 5c. e) Microelectrode CVs of anolyte and catholyte reservoirs of the redox flow batteries shown in Figure 5c before and after 100 cycles at 125 mM. f) Charge-discharge curves of the 125 mM cycling shown in Figure 5c.

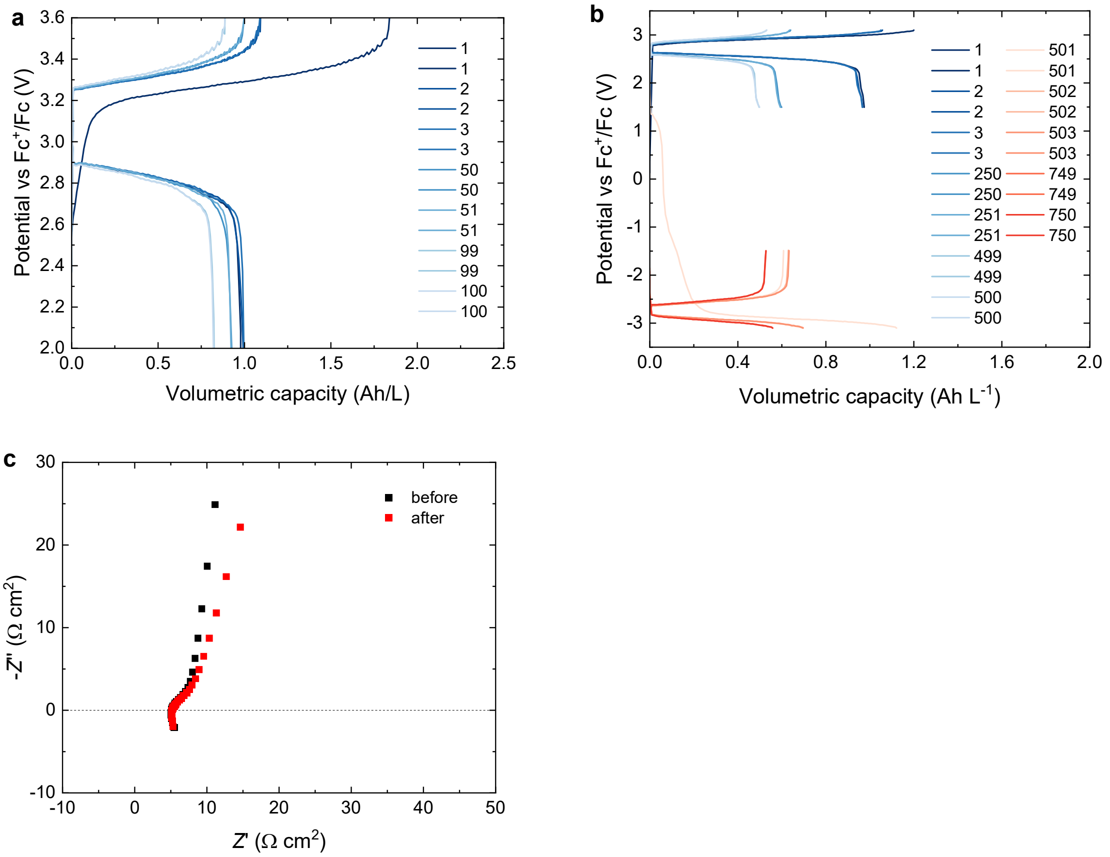

Figure S12. a) Charge-discharge curves of the cycling shown in Figure 6b. b) Charge-discharge curves of the cycling shown in Figure 6d. c) PEIS measurements before and after the flow cell cycling shown in Figure 6d.

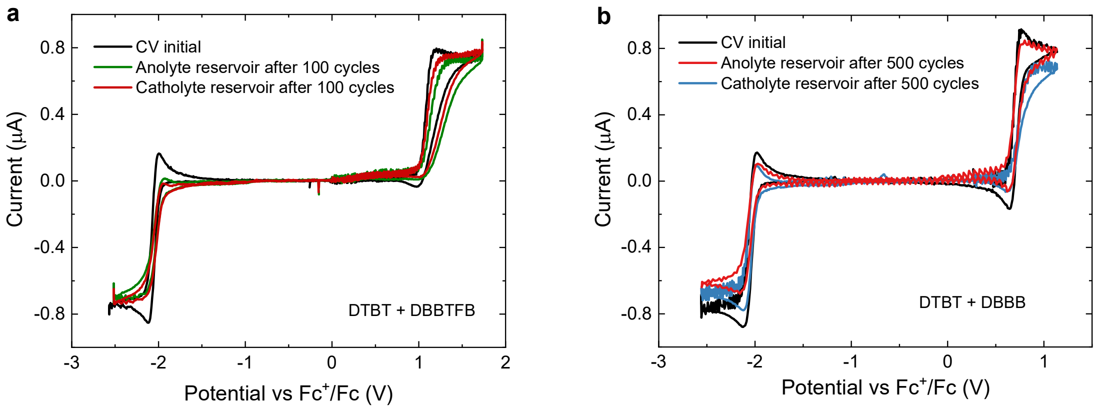

Figure S13. a) Microelectrode CVs of anolyte and catholyte reservoirs of a redox flow battery containing a mixed solution of **DTBT** (50 mM) with **DBBTFB** (50 mM) in acetonitrile with $TBAPF_6$ (300 mM) before and after 100 cycles of operation. b) Microelectrode CVs of the anolyte and catholyte reservoirs of a redox flow battery containing a mixed solution of **DTBT** (50 mM) with **DBBB** (55 mM) in acetonitrile with $TBAPF_6$ (200 mM) before and after 500 cycles of operation. The redox flow batteries used a porous Daramic 175 membrane separator.

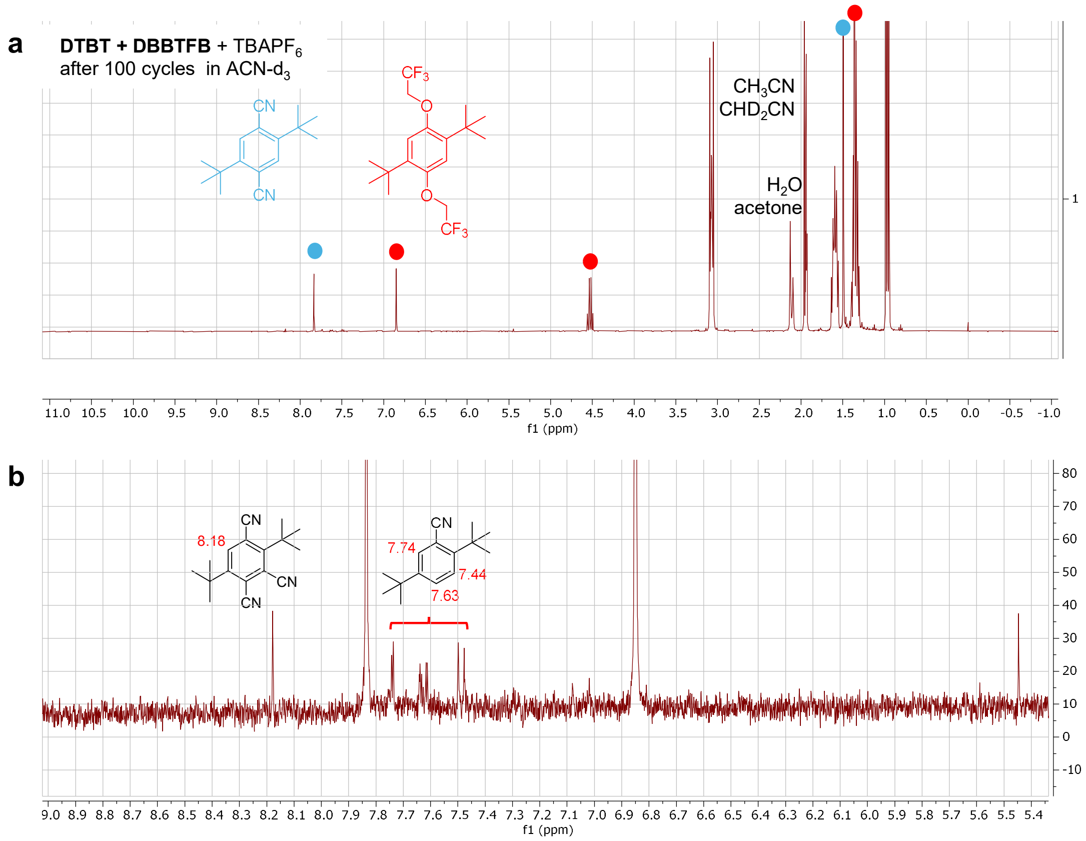

Figure S14. NMR spectra of **DTBT** + **DBBTFB** before and after redox flow battery cycling. a) ^{1}H NMR spectrum of **DTBT** + **DBBTFB** after 100 cycles of operation. Signals that are not assigned are from TBA^+ ions. b) Zoom-in of ^{1}H NMR spectrum of **DTBT** + **DBBTFB** after 100 cycles of operation. Two possible (but unconfirmed) assignments for degradation products are shown. The signal at 5.45 ppm is from a trace of dichloromethane.

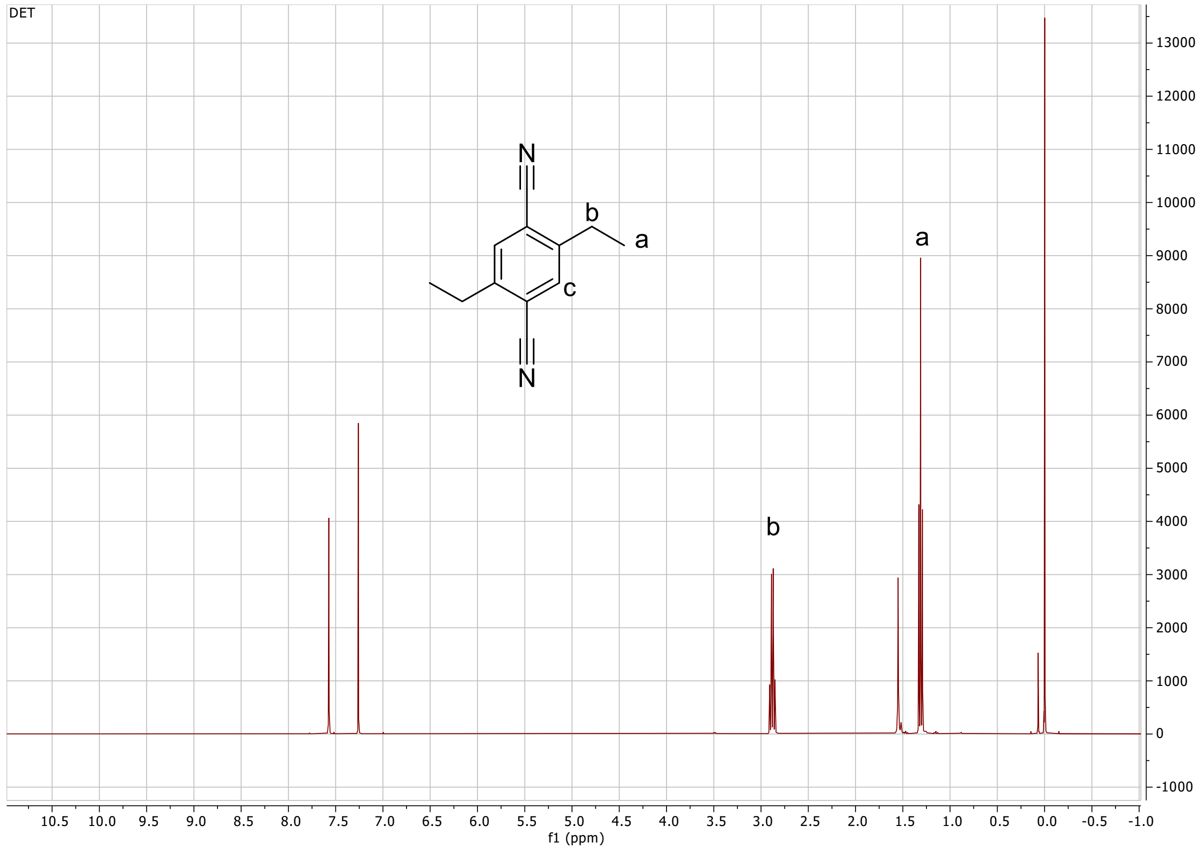

Figure S15. ^{1}H NMR spectrum of DET in $CDCl_3$. Solvent, TMS, and impurity signals are at 0 ppm (TMS), 0.07 ppm (silicon grease),1.56 ppm (H_2O), and 7.26 ppm ($CHCl_3$).

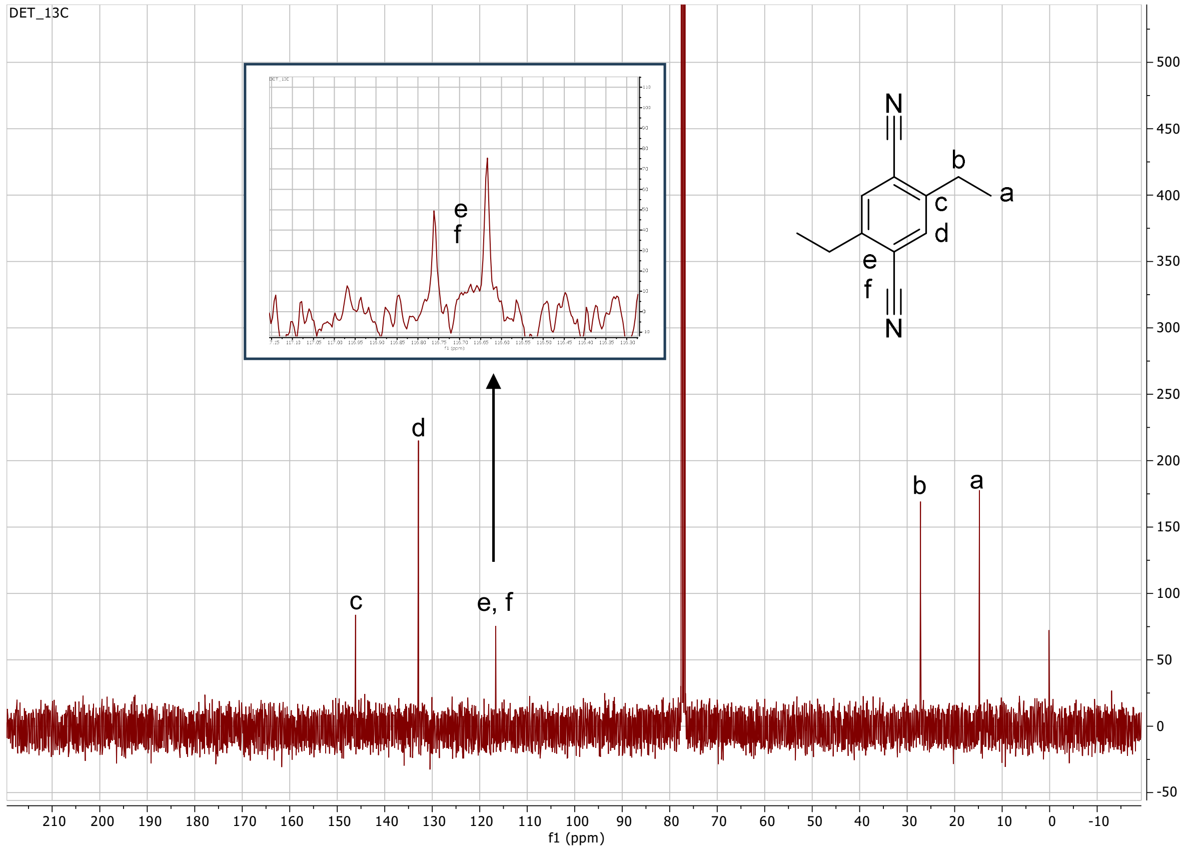

Figure S16. ^{13}C NMR spectrum of DET in $CDCl_3$. The inset shows the two peaks at 116.76 and 116.63 ppm of the quaternary carbons e and f. Signals at 0 and 77.16 ppm are from TMS and $CDCl_3$.

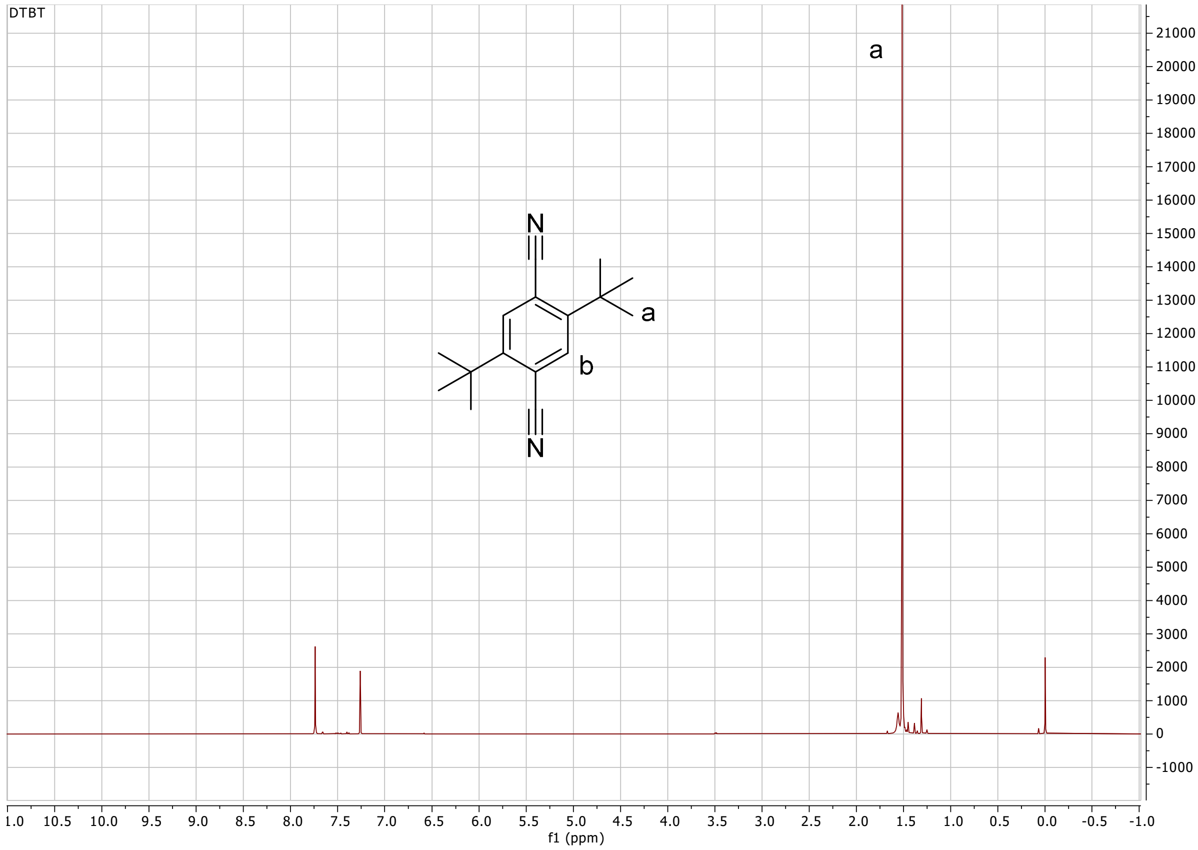

Figure S17. ^{1}H NMR spectrum of DTBT in $CDCl_3$. Solvent, TMS, and impurity signals are at 0 ppm (TMS), 0.07 ppm (silicon grease),1.56 ppm (H_2O), and 7.26 ppm ($CHCl_3$).

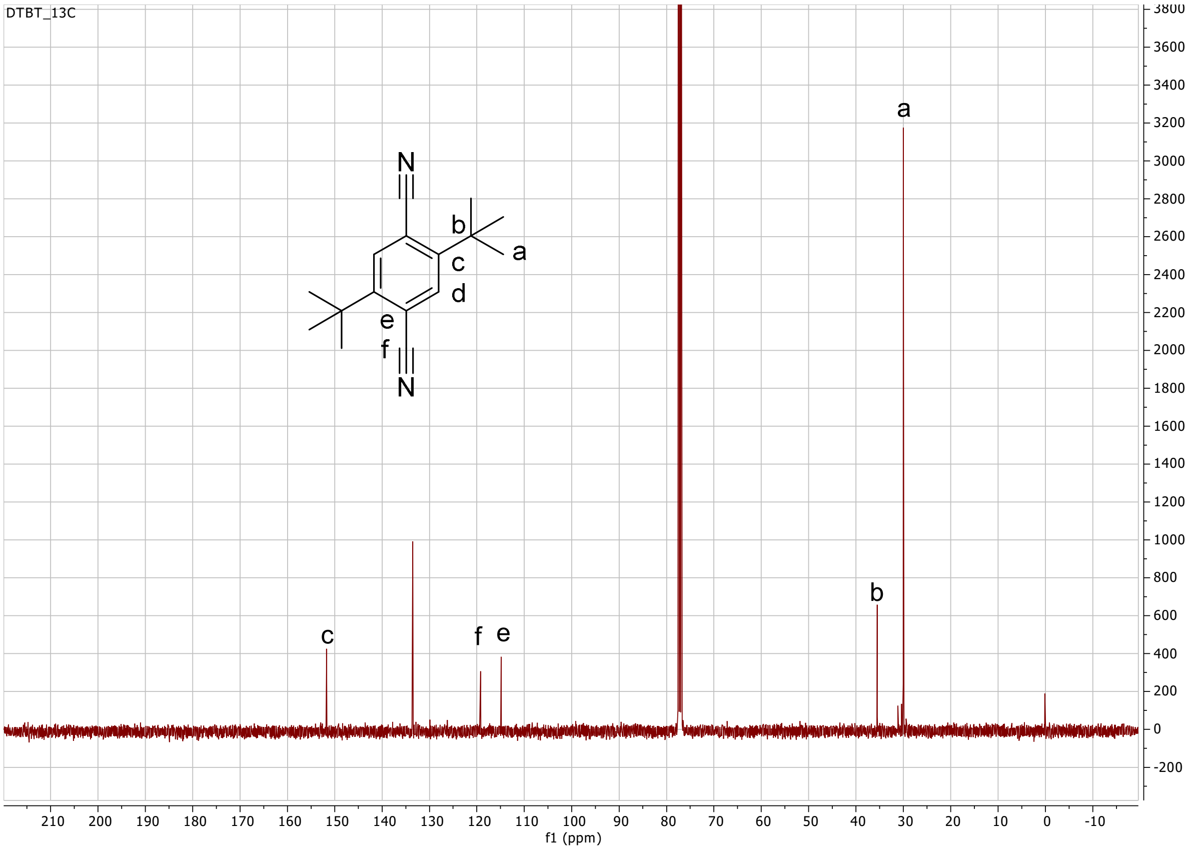

Figure S18. ^{13}C NMR spectrum of DTBT in $CDCl_3$. Signals at 0 and 77.16 ppm are from TMS and $CDCl_3$.

Supplementary references

(S1) Sugamata, K.; Kobayashi, S.; Iihama, T.; Minoura, M. Gas Adsorption in R_2-MOF-5 Difunctionalized with Alkyl Groups. *Eur. J. Inorg. Chem.* **2021**, 3185−3190.

(S2) Yu, C.; Bourrelly, S.; Martineau, C.; Saidi, F.; Bloch, E.; Lavrard, H.; Taulelle, F.; Horcajada, P.; Serre, C.; Llewellyn, P. L.; Magnier, E.; Devic, T. Functionalization of Zr-based MOFs with alkyl and perfluoroalkyl groups: the effect on the water sorption behavior. *Dalton Trans.* **2015**, *44*, 19687−19692.

(S3) Reck, C. E.; Winter, C. H. Synthesis and Characterization of a Dimagnesiated Aromatic Compound: Oligomeric Derivatives of 1,4-Bis(chloromagnesio)-2,5-di-*tert*-butylbenzene. *Organometallics* **1997**, *16*, 4493−4496.

(S4) Mella, M.; Fasani, E.; Albini, A. Electron Transfer Photoinduced Cleavage of Acetals. A Mild Preparation of Alkyl Radicals. *J. Org. Chem.* **1992**, *57*, 3051−3057.

(S5) Wang, C.; Russell, G. A.; Trahanovsky, W. S. Homolytic Base-Promoted Aromatic Alkylations by Alkyl Halides. *J. Org. Chem.* **1998**, *63*, 9956−9959.

(S6) Bheemireddy, S. R.; Li, Z.; Zhang, J.; Agarwal, G.; Robertson, L. A.; Shkrob, I. A.; Assary, R. S.; Zhang, Z.; Wei, X. Cheng, L.; Zhang, L. Fluorination Enables Simultaneous Improvements of a Dialkoxybenzene-Based Redoxmer for Nonaqueous Redox Flow Batteries. *ACS Appl. Mater. Interfaces* **2022**, *14*, 28834− 28841.

(S7) Yan, Y.; Robinson, S. G.; Sigman, M. S.; Sanford, M. S. Mechanism-Based Design of a High-Potential Catholyte Enables a 3.2 V All-Organic Nonaqueous Redox Flow Battery. *J. Am. Chem. Soc.* **2019**, *141*, 15301–15306.

(S8) Pancoast, A. R.; McCormack, S. L.; Galinat, S.; Walser-Kuntz, R.; Sigman, M. S. Data science enabled discovery of a highly soluble 2,2′-bipyrimidine anolyte for application in a flow battery. *Chem. Sci.* **2023**, *14*, 13734–13742.

(S9) Xing, X.; Liu, Q.; Xu, W.; Liang, W.; Liu, J.; Wang, B.; Lemmon, J. P. All-Liquid Electroactive Materials for High Energy Density Organic Flow Battery. *ACS Appl. Energy Mater.* **2019**, *2*, 2364–2369.

(S10) Huo, Y.; Xing, X.; Zhang, C.; Wang, X.; Li, Y. An all organic redox flow battery with high cell voltage. *RSC Adv.* **2019**, *9*, 13128–13132.

(S11) Wang, X.; Xing, X.; Huo, Y.; Zhao, Y; Chen, H. Study of Tetraethylammonium bis(trifluoromethylsulfonyl)imide as a Supporting Electrolyte for an All-organic Redox Flow Battery Using Benzophenone and 1,4-di-tert-butyl-2,5-dimethoxybenzene as Active Species. *Int. J. Electrochem. Sci.* **2018**, *13*, 6676–6683.

(S12) Xing, X.; Liu, Q.; Wang, B.; Lemmon, J. P.; Xu, W. Q. A low potential solvent-miscible 3-methylbenzophenone anolyte material for high voltage and energy density all-organic flow battery. *J. Power Sources* **2020**, *445*, 227330.

(S13) Liu, Y.; Dai, G.; Chen, Y.; Wang, R.; Li, H.; Shi, X.; Zhang, X.; Xu, Y.; Zhao, Y. Effective Design Strategy of Small Bipolar Molecules through Fused Conjugation toward 2.5 V Based Redox Flow Batteries. *ACS Energy Lett.* **2022**, *7*, 1274–1283.

(S14) Xu, D., Zhang, C., Zhen, Y., Zhao, Y. & Li, Y. A high-rate nonaqueous organic redox flow battery. *J. Power Sources* **2021**, *495*, 229819.

(S15) Duan, W.; Huang, J. H.; Kowalski, J. A.; Shkrob, I. A.; Vijayakumar, M.; Walter, E.; Pan, B. F.; Yang, Z.; Milshtein, J. D.; Li, B.; Liao, C.; Zhang, Z. C.; Wang, W.; Liu, J.; Moore, J. S.; Brushett, F. R.; Zhang, L.; Wei, X. L "Wine-Dark Sea" in an Organic Flow Battery: Storing Negative Charge in 2,1,3-Benzothiadiazole Radicals Leads to Improved Cyclability. *ACS Energy Lett.* **2017**, *2*, 1156–1161.

(S16) Daub, N.; Janssen, R. A. J.; Hendriks, K. H. Imide-Based Multielectron Anolytes as High-Performance Materials in Nonaqueous Redox Flow Batteries. *ACS Appl. Energy Mater.* **2021**, *4*, 9248–9257.

(S17) Ahn, S.; Son, M.; Singh, V.; Yun, A.; Baik, M.-H.; Byon, H. R. Stabilization of Naphthalene Diimide Anions by Ion Pair Formation in Nonaqueous Organic Redox Flow Batteries. *J. Am. Chem. Soc.* **2024**, *146*, 4521– 4531.
